# Sustainable Olive Pomace Extracts for Skin Barrier Support

**DOI:** 10.3390/pharmaceutics17080962

**Published:** 2025-07-25

**Authors:** Roberta Cougo Riéffel, Lucas Agostini, Naira Poener Rodrigues, Simone Jacobus Berlitz, Lígia Damasceno Ferreira Marczak, Irene Clemes Külkamp-Guerreiro

**Affiliations:** 1 Programa de Pós-graduação em Ciências Farmacêuticas, Universidade Federal do Rio Grande do Sul, Porto Alegre 90610-000, Brazil; roberta.rieffel@ufrgs.br (R.C.R.);; 2 Programa de Pós-graduação em Engenharia Química, Universidade Federal do Rio Grande do Sul, Porto Alegre 90610-000, Brazil; agostinilucas01@hotmail.com (L.A.); nairapoernersa@gmail.com (N.P.R.); ligia@enq.ufrgs.br (L.D.F.M.)

**Keywords:** antioxidant, extraction, olive byproduct, phenolic compounds, skin barrier homeostasis

## Abstract

**Background**: Olive pomace, a byproduct of olive oil production, represents approximately 85% of the processed material and poses environmental risks when improperly discarded. Its composition is rich in polyphenols with potential for cosmetic use, especially in skin barrier care. **Objective**: To develop a natural extract rich in antioxidants from olive pomace using sustainable solvents (water and 1,3-propanediol) for skin barrier support. **Methods**: The phenolic composition and in vitro biological activities of the extracts were analyzed. **Results**: The extracts demonstrated a reducing capacity (15 to 33 mg GAE/g) and flavonoid content (4 to 5 mg QE/g). In addition, their antioxidant capacity was proven through the inhibition of the DPPH radical (7% to 91%) and ABTS (7% to 95%) and the reduction in oxidation in the beta-carotene/linoleic acid system (6% to 35%), presenting results superior to those of tocopherol acetate. The hydroxytyrosol and oleuropein compounds, ranging from 28 to 54 and 51 to 85 µg/mL, respectively, were quantified via HPLC. The extract with the highest levels of hydroxytyrosol and oleuropein was analyzed via UHPLC-QqTOF-MS, and 33 compounds were identified. This extract showed antiglycation activity (24% to 40%). The incorporation of this extract into a cosmetic emulsion resulted in sufficient antioxidant capacity to replace tocopherol acetate. **Conclusions**: The use of effective extraction techniques and nontoxic solvents ensures the sustainability and safety of the extract for application as a natural cosmetic ingredient, aiming to promote the health and integrity of the skin barrier.

## 1. Introduction

The skin, the outermost layer of the human body, performs essential functions such as regulating temperature, preventing transepidermal water loss (TEWL), controlling permeability to electrolytes and other substances, and participating in the immune and endocrine systems. It also acts as a physical barrier against external aggressors and ultraviolet radiation. The epidermal permeability barrier, which is mainly composed of the stratum corneum (SC), is the primary defense for maintaining hydration, skin homeostasis, and, consequently, the survival of organisms [1,2]. The SC is a multilayered structure formed by anucleated and flattened keratinocytes organized in highly ordered lipid lamellae. Alterations in the SC compromise the integrity of the skin barrier, making the maintenance of SC homeostasis essential for skin health [1].

Constant exposure of the skin to environmental factors such as UV radiation, diet, and pollution stimulates the production of reactive oxygen species (ROS) [3]. These species include free radicals, nonradical species, and reactive nitrogen species (RNS), which interact with proteins, lipids, and DNA, causing cellular and structural damage in the skin matrix [4,5]. Oxidative stress occurs when there is an imbalance between ROS production and the antioxidant system’s capacity to neutralize them [4]. Skin defense against this stress involves antioxidant enzymes such as glutathione, catalase, superoxide dismutase, and peroxidase, as well as nonenzymatic antioxidant molecules [6]. The intrinsic antioxidant system often demonstrates insufficient capacity for the complete neutralization of reactive oxygen species (ROS); consequently, its deficiency necessitates the exogenous administration or topical application of natural antioxidants, such as phenolic compounds [3,6,7]. Strengthening this system can effectively protect the skin barrier, as lipid and protein oxidation compromises its integrity, triggering various pathologies [2,6].

Phenolic compounds directly eliminate or reduce ROS generation by forming relatively stable radicals that interrupt the cellular oxidative chain. Furthermore, they enhance the cellular antioxidant capacity by activating nuclear factor erythroid 2-related factor 2 (Nrf2) [3]. Topical application of polyphenols improves skin barrier function by increasing the expression of structural proteins such as filaggrin, involucrin, and loricrin. TEWL also occurs through the activation of aquaporin 3 (AQP3), which enhances stratum corneum hydration. The antioxidant action of polyphenols contributes to cellular longevity through signaling pathways, including mitogen-activated protein kinase/extracellular signal-regulated kinase (MPK-1/ERK), silent information regulator 2 homolog 1/abnormal Dauer Formation protein 16 (SIR-2.1/DAF-16), and insulin-like growth factor 1 (IGF-1) [8].

Given the potential of polyphenols in skin barrier repair, sustainable sources of these compounds, such as agricultural byproducts, have attracted increasing interest [9]. Olive pomace, a solid waste byproduct of olive oil processing, presents a promising opportunity for the development of innovative and sustainable products. This pomace can be utilized as a raw material for extracting bioactive compounds relevant to the cosmetic sector, thereby contributing to a circular ecosystem of value generation [10,11]. Compared to olive pomace, which accounts for approximately 85% of the total mass from olive production, olive pomace necessitates appropriate disposal to mitigate its potential environmental impacts [12]. The implementation of sustainable management practices for this byproduct can help reduce waste generation while maximizing the economic potential of olive oil production [13,14]. The potential reutilization of olive pomace is largely attributed to its phenolic composition, as approximately 97% of these compounds remain in the pomace [15]. Polyphenol-rich plant extracts, including those derived from olive pomace, hold promise for enhancing skin barrier homeostasis and promoting overall skin health [6]. Several strategies are currently being explored to optimize the recovery and valorization of olive phenolic compounds [12].

Emerging technologies, such as supercritical fluid extraction and pulsed electric field extraction, have been explored; however, their cost-effectiveness may limit their application [16,17]. Conventional extraction techniques use organic solvents, which have several drawbacks related to their flammability, non-degradability, and toxic nature, increasing safety concerns [18]. In this context, solid–liquid extraction techniques utilizing nontoxic solvents have gained increasing recognition for their applicability in various industries. Among these, 1,3-propanediol—a naturally derived solvent obtained through corn fermentation—has emerged as a viable alternative to synthetic and potentially harmful options [19]. Its incorporation into cosmetic formulations represents a significant advancement, offering both efficacy and safety while enhancing the interaction between bioactive compounds and the skin. Additionally, 1,3-propanediol confers physiological benefits, notably reducing TEWL and improving skin hydration, both of which are fundamental to maintaining skin barrier integrity [20,21].

Recent studies have highlighted the rich phenolic composition and multifunctional bioactivity of extracts derived from olive byproducts for cosmetic applications. Cádiz-Gurrea et al. [22] identified 49 phenolic compounds in olive leaf extracts, which demonstrated synergistic antioxidant, anti-inflammatory, photoprotective, and antiaging properties relevant to skin health. Similarly, Kishikawa et al. [23] evaluated extracts from olive oil byproducts, including leaves, stems, flowers, pomace, fruit pulp, and seeds, confirming their biological activity for skin care through effects such as a reduction in melanin biosynthesis, the stimulation of collagen production, and anti-inflammatory responses. Despite the large number of olive-based cosmetic products available on the market, formulations utilizing olive pomace remain scarce. Rodrigues et al. [24] emphasized this gap, highlighting the importance of exploring the phenolic profile and potential applications of olive pomace in cosmetic development.

The valorization of olive residues aligns with the United Nations (UN) Sustainable Development Goals (SDGs), specifically SDG 12, which promotes responsible consumption and production by encouraging prevention, recycling, and reuse to minimize environmental impacts. Additionally, this approach contributes to SDG 9 by fostering innovation and sustainable industrialization, SDG 13 by reducing environmental impacts and mitigating climate change, and SDG 3 by supporting the development of natural ingredients that promote health and well-being [25]. Additionally, consumers increasingly value upcycling in the cosmetics sector, and transforming raw materials that were previously discarded into high-value products enhances the sustainable profile of final formulations. Byproducts, once regarded as waste, are now attracting considerable interest because their composition is rich in bioactive compounds [24].

To address the inherent difficulties of extraction methods utilizing organic solvents, this study aimed to develop a natural and innovative olive pomace extract for topical application. This endeavor involved an effective, simple, economical, and sustainable process for extracting phenolic compounds from the plant matrix, leveraging a natural and skin-compatible solvent in accordance with green chemistry principles. The resulting extracts were comprehensively characterized for their phytochemical composition, in vitro antioxidant capacity, and antiglycation activity. The extract was subsequently integrated into a cosmetic emulsion, revealing the profound implications of such activities for skin health and barrier integrity.

## 2. Materials and Methods

### 2.1. Reagents, Chemicals, and Equipment

Reagents and chemicals: Folin–Ciocalteu phenol reagent, oleuropein, hydroxytyrosol, 2,2-diphenyl-1-picrylhydrazyl (DPPH), 2,2′-azino-bis (3-ethylbenzothiazoline-6-sulfonic acid) diammonium salt (ABTS), gallic acid, 6-hydroxy-2,5,7,8-tetramethylchromane-2-carboxylic acid (Trolox^®^), bovine serum albumin, and β-carotene were obtained from Sigma–Aldrich, (St. Louis, MO, USA) Potassium persulfate and fructose were obtained from Synth (Diadema, Brazil). Ethanol and methanol were obtained from Exôdo Científica (Sumaré, Brazil). Linoleic acid, acetonitrile, and glacial acetic acid were obtained from Vetec (Duque de Caxias, Brazil). Tween 20 was obtained from Dinâmica (Indaiatuba, Brazil). Cloroform, carnosine, and tocopheryl acetate were obtained from Neon (Suzano, Brazil), Chemyunion (Sorocaba, Brazil) and Basf (Ludwigshafen, Germany), respectively.

Equipment: UV–VIS spectrophotometer T 80+, PG Instruments Limited (Leicester, UK); microplate reader SpectraMax^®^ M5 Series Multi-Mode Microplate Readers, Molecular Devices LLC (São José, CA, USA); UHPLC-QqTOF-MS Impact II Bruker Daltonics Inc. (Billerica, MA, USA); and HPLC Shimadzu CBM-20A System (Shimadzu Europa GmbH, Duisburg, Germany).

### 2.2. Olive Pomace Collection

The olive pomace from the 2022 harvest (Arbequina, Arbosana, Picual, and Koroneike cultivars) was collected from an agroindustry located in the Campanha Region, Rio Grande do Sul, Brazil. Olive pomace was obtained from the two-phase centrifugation system immediately after olive oil extraction.

### 2.3. Olive Pomace Extraction Process

The samples were dried in a forced-air oven at a temperature of 55 °C (parameter defined in previous analyses). After drying, the samples were ground in a knife mill and homogenized into particles (≤400 µm). The solid–liquid extraction method was employed for 20 min under continuous stirring at 55 °C. Different solvents and solid/liquid ratios were investigated to prepare three different olive pomace extracts. Olive pomace extract 1 (OPE1) was prepared with water as the solvent and a 1:15 (*w*/*v*) solid/liquid ratio. Olive pomace extract 2 (OPE2) was developed with 1,3-propanediol/water (50:50, *v/v*) as the solvent and a 1:15 (*w/v*) solid/liquid ratio. Olive pomace extract 3 (OPE3) was prepared with 1.3-propanediol and a 1:10 (*w/v*) solid/liquid ratio. The olive pomace extracts were subsequently vacuum-filtered.

### 2.4. Reducing Capacity of the Folin–Ciocalteu Reagent

The reducing capacity of the Folin–Ciocalteu reagent was determined on the basis of the methodology described by Waterhouse [26]. Briefly, a gallic acid concentration curve (10 to 90 mg/L) was constructed (R^2^ = 0.9937). The results are expressed in milligrams of gallic acid per gram of dried olive pomace (mg GAE/g). The reaction mixture consisted of 850 μL of distilled water, 200 μL of sample, and 100 μL of Folin–Ciocalteu reagent. The mixture was vortexed, and 850 μL of 7% sodium carbonate was added. The solution was kept in the dark for 1 h. The absorbance was measured at 765 nm with a UV–VIS spectrophotometer.

### 2.5. Total Flavonoid Content

The total flavonoid content methodology was based on the technique described by Chang et al. [27]. The samples (0.5 mL) were mixed with 1.5 mL of water and 0.1 mL of 10% aluminum trichloride (AlCl). Then, 0.1 mL of 1 M potassium citrate and 2.8 mL of distilled water were added. The samples were incubated for 30 min in the dark, and the absorbance was measured at 415 nm via a UV–VIS spectrophotometer. A blank control was prepared in the same way without the addition of the sample or aluminum chloride. A quercetin concentration curve was prepared (R^2^ = 0.9955). The results are expressed as milligrams of QE per gram of dried olive pomace (mg QE/g).

### 2.6. Deactivation of the 2,2′-Azinobis-3-Ethylbenzothiazoline-6-Sulfonic Acid (ABTS) Radical

The ABTS radical deactivation methodology was adapted from the protocol of Re et al. [28]. For the formation of the ABTS radical, 5 mL of 14 mM ABTS solution was mixed with 5 mL of 4.9 mM potassium persulfate and kept at room temperature, protected from light, for 16 h. For the reaction system, 0.3 mL of ABTS was added to 30 mL of ethanol. The absorbance was measured at 750 nm in a UV–VIS spectrophotometer. Ethanol was used as a blank. Initially, 1.8 mL of the working ABTS solution was analyzed in spectrophotometric mode. Then, 0.2 mL of the test mixture was added, and the mixtures were homogenized and kept in the dark for 60 min. The absorbances were measured in spectrophotometric mode. Different concentrations of OPE1, OPE2, and OPE3 were evaluated (5, 7.5, and 10 mg/mL were diluted in ethanol). The natural antioxidant tocopheryl acetate and the synthetic antioxidant butylated hydroxytoluene (BHT) were analyzed for comparison. The percentage of inhibition was calculated via Equation (1):

(1) %inhibition=((Abs.initial−Abs.final)/Abs.initial)×100

where Abs.initial is the absorbance of the radical before the addition of the sample and Abs.final is the absorbance of the radical after analysis in kinetic mode. The results are expressed as percentages of ABTS inhibition and as IC_50_ values, which were defined as the antioxidant concentration required to reduce the absorbance of the control by 50%.

### 2.7. Antioxidant Capacity Determination via DPPH Radical Analysis

The determination of antioxidant activity via the DPPH radical assay followed the methodology described by Gonzales et al. [29]. Three different concentrations (0.25 to 100 mg/mL) of OPE1, OPE2, and OPE3 were evaluated. The natural antioxidant tocopheryl acetate (0.25 to 100 mg/mL) and the synthetic antioxidant butylated hydroxytoluene (BHT) (0.05 to 1.00 mg/mL) were analyzed for comparison. The results are expressed as percentages of DPPH inhibition (5, 7.5, and 10 mg/mL concentrations) and as IC_50_ values, which were defined as the antioxidant concentration required to reduce the absorbance of the control by 50%. A 20 μg/mL DPPH solution was prepared in methanol. For the reactions, 3 mL of the DPPH reagent was mixed with 1 mL of the sample or with methanol as a negative control. The mixtures were homogenized and kept in the dark for 2 h. The absorbances were measured at 515 nm via a UV–VIS spectrophotometer. The percentage of inhibition was calculated via Equation (2):

(2) $$\%Deactivation=\frac{Ac-As}{Ac}\times 100$$

where Ac is the absorbance of the control and As is the absorbance of the sample.

### 2.8. Antioxidant Capacity of the Beta-Carotene/Linoleic Acid System

The antioxidant capacity of the beta-carotene/linoleic acid system followed the methodology described by Paim et al. [30]. A reaction mixture containing 60 mg of linoleic acid, 600 mg of Tween 20, and 6 mg of beta-carotene was prepared. Chloroform (30 mL) was used for beta-carotene solubilization, and it was removed via rotary evaporation (Fisaton Rotary Evaporator, Model 450, Brazil). Subsequently, approximately 150 mL of distilled water saturated with oxygen was added to the mixture under constant stirring. Dilutions were made at concentrations of the extracts of 1.0 and 2.0 mg/mL. Tocopheryl acetate was used as a positive control. Beta-carotene/linoleic acid solution (2.5 mL) was added to 0.2 mL of each sample. The absorbance was measured via a UV–VIS spectrophotometer at 470 nm immediately after sample preparation. The tubes were kept in a water bath at 50 °C and exposed to light. The second reading was performed after 120 min. The results are expressed as the percentage of inhibition of beta-carotene oxidation. The decrease in the absorbance of the samples was used to determine the percentage of oxidation of the olive pomace extracts via Equation (3):

(3) %Oxidation inhibition=[1−(Abs.ini−Abs/Abs.cont.ini−Abs.cont.)]×100

where Abs.ini is the absorbance of the sample at the initial reaction time, Abs. represents the absorbance of the sample after 120 min, Abs.cont.ini. is the absorbance of the negative control at the initial time, and Abs. cont. is the absorbance of the negative control after 120 min.

### 2.9. Stability Study

The stability of the olive pomace extracts was analyzed following the methodology described by Kesente et al. [31]. The samples were stored under different conditions: refrigerated (5 °C), at room temperature (25 °C), and in a climate chamber (40 °C). The samples were analyzed at 0, 30, 60, and 90 days for their Folin–Ciocalteu reducing capacity (described in Section 2.4).

### 2.10. Simultaneous Determination of Hydroxytyrosol and Oleuropein via HPLC

OPE1, OPE2, and OPE3 were analyzed as described by Habibi et al. [32], using a Shimadzu CBM-20A System equipped with an SIL-20AC automatic sampler, a CTO-20AC column oven, and an SPD-20AV UV/VIS detector. The data were acquired and analyzed via LC-Solution software. The column used was a Synergi Hydro-RP C18 4 μm, 250 × 4.6 mm, coupled to an ODS-C18 precolumn, 5 μm, 4 × 3 mm (Phenomenex, California, USA). The mobile phase was water with glacial acetic acid (0.5%) (eluent A) and acetonitrile (eluent B). The elution gradient used was 5–20% B (0–30 min), 20–30% B (30–40 min), and 30–35% B (40–45 min), with a flow rate of 1 mL.min^−1^. The column temperature was maintained at 25 °C, and the injection volume was 20 μL. The OPE2 and OPE3 samples were diluted 5-fold in ultrapure water, and OPE1 was not diluted. The detection wavelength was 280 nm. An analytical curve of hydroxytyrosol and oleuropein was constructed (R^2^= 0.9999).

### 2.11. Qualitative Analysis Via Ultrahigh-Efficiency Liquid Chromatography Coupled with Ultrahigh-Resolution Mass Spectrometry (QTOF) (UHPLC-QqTOF-MS)

The methodology was based on the technique described by Peralbo-Molina et al. [33]. The OPE3 extract was selected for evaluation via UHPLC-QqTOF-MS. The sample was prepared by 10-fold dilution in ultrapure water, followed by a filtration step (0.22 μm). For the analysis, a volume of 10 μL of the sample was injected at a flow rate of 0.35 mL/min. The mobile phase used was a 0.1% (*v*/*v*) aqueous solution of formic acid (phase A) and pure acetonitrile (phase B). The analyte separation was carried out via a Kinetex 1.7 µm EVO C18 A column (100 × 42.1 mm internal diameter, 5 µm particle size; Phenomenex, California, USA) in the temperature range of 40–60 °C. The gradient method was as follows: 95% A (4 min), 2% A (35 min), 2% A (40 min), 95% A (44 min), and 0% A (50 min). The dual ESI source was operated in negative ionization mode with the following conditions: nebulizer gas at 4.0 bar and the flow rate and drying gas temperature at 9.0 L/min and 200 °C, respectively. The capillary voltage was set at 2500 V, and the end plate offset was adjusted to 500 V. The bbCID (MS-MS/MS) scanning mode was used, and the collision energies were set to scan automatically from 20 to 60 eV. The full scan was performed at a rate of one spectrum per second within the range of 50 to 1500 *m*/*z*. Data analysis and processing were carried out via Data Analyses software (Bruker Daltoniks, MA, USA). The precision limit for elemental composition confirmation was set at 5 ppm. To confirm the presence of compounds in the extract, mSigma data, the MassBank Data Platform (https://massbank.eu/MassBank/search, accessed in January 2022 and January–February 2023), and information from the scientific literature were used, considering parameters such as fragment ions, molecular formula, and molecular mass.

### 2.12. Antiglycation Activity

OPE3 antiglycation activity was evaluated via the BSA–fructose model [34]. Reaction solutions of fructose (1.5 mol/L), bovine serum albumin (BSA) (60 mg/mL), and carnosine (150 µg/mL) were prepared in potassium phosphate buffer (0.2 mol/L, pH 7.4) containing 0.06% sodium azide. The test solution was prepared by diluting 0.2 and 3.5 mg/mL OPE3 extract in the same buffer. A total of 500 μL of the fructose solution was incubated with 500 μL of the test sample solution at 37 °C. After 2 h, 500 μL of the BSA solution was added, and the fluorescence was measured at time zero. The samples were returned to the incubator at 37 °C for 7 days. The fluorometric assays were performed with a microplate reader at an emission wavelength of 450 nm with excitation at 340 nm. A negative control was prepared using phosphate buffer, fructose, and BSA. The glycation inhibition percentage was calculated via Equation (4):

(4) $$\%Inhibition=1-(\frac{\left(Fi7d\;sample-Fi0d\;sample\right)}{\left(Fi7d\;neg\;cont-Fi0d\;neg\;cont\right)})\times 100$$

where Fi0d and Fi7d represent the fluorescence intensity at time zero and after 7 days of reaction, respectively; and neg cont refers to the negative control.

### 2.13. Antioxidant Capacity of Cosmetic Formulations

The OPE3 extract was selected for incorporation into a cosmetic emulsion prepared via the phase inversion method [35]. Table 1 presents the qualitative and quantitative compositions of the emulsions and the respective functions of each component. OPE3 (1% *w*/*w*) was incorporated as the active ingredient; and a control formulation was prepared in which the extract was replaced with tocopheryl acetate (1% *w*/*w*) for comparison. The pH of the formulations was adjusted to 5 with citric acid. The antioxidant activities of the cosmetic formulations were analyzed via the DPPH radical scavenging method, as described in Section 2.8. The cosmetic formulation without the active ingredients was also evaluated for comparison.

### 2.14. Statistical Analysis

Statistical analyses were performed via ANOVA followed by Tukey’s or Dunnett’s test in GraphPad Prism version 5.0 software. The data are expressed as the means ± standard deviations. A *p* value of <0.05 was considered statistically significant. All tests were performed in triplicate.

## 3. Results and Discussion

### 3.1. Olive Pomace Extraction

Table 2 presents the reducing capacity of the Folin–Ciocalteu reagent and the total flavonoid content.

The extraction of olive pomace was developed by applying the following green chemistry principles: safer solvents, design for economic efficiency, and the use of renewable raw materials [36,37]. Water and 1,3-propanediol were used as environmentally friendly solvents (as an alternative to harmful organic solvents).

Extracts using 1,3-propanediol (OPE2 and OPE3) showed greater reducing capacities than the aqueous extract (OPE1), highlighting its potential as a green solvent for extracting valuable compounds from olive pomace. This result reinforces the importance of selecting extraction conditions that combine efficiency and sustainability. Indeed, overcoming the limitations of conventional extraction processes requires the use of innovative solvents that are environmentally sustainable, less toxic, economically viable, and more selective. Additionally, factors such as the plant matrix characteristics, solubility, molecular structure, and chemical nature of bioactive compounds must be carefully considered to ensure maximum extraction efficiency [38]. The reducing capacity observed for the aqueous extract (OPE1) aligns with values reported in the literature for similar extraction methods. For example, Goldsmith et al. [37] obtained reducing capacities of 13.76 ± 0.91 mg GAE/g and 19.71 ± 1.41 mg GAE/g via conventional and ultrasound-assisted water extraction, respectively. Other solvents have also been explored: Almeida Pontes et al. [39] achieved a relatively high reducing capacity of 25.32 mg ± 0.35 GAE/g using a 50:50 ethanol/water mixture. Furthermore, Chanioti and Tzia [40] investigated green solvents such as natural deep eutectic solvents (NADESs) and reported reducing capacities ranging from 13 to 34 mg GAE/g.

According to Chang et al. [27], the aluminum chloride colorimetric method, which is specific for flavonols and flavones, could underestimate the content of other classes of flavonoids in samples with many compounds. This limitation may explain the low total flavonoid contents presented in Table 2. The results for total flavonoid content followed a similar pattern to those observed for reducing capacity. Compared to the aqueous extract, the extracts prepared with 1,3-propanediol as the solvent presented a significantly greater content of total flavonoids. Furthermore, the extract produced solely with 1,3-propanediol presented the highest flavonoid content among all the extracts analyzed.

Overall, the results of this investigation clearly demonstrate the efficacy of the proposed sustainable extraction process for obtaining bioactive compounds from olive pomace. In particular, the use of 1,3-propanediol as a solvent was highly effective, yielding extracts with superior antioxidant properties. Thus, these extracts are promising alternatives for skin care, especially for maintaining skin barrier homeostasis, since, according to Butkeviciute et al. [5], plant-based phenolic compounds demonstrate, in addition to their antioxidant capacity and low toxicity, excellent skin permeability properties.

### 3.2. ABTS and DPPH Radical Analysis of the Antioxidant Capacity

**Figure 1:**
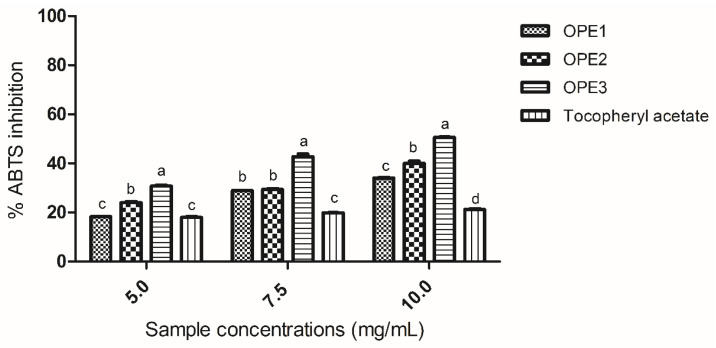
The results of the analysis of antioxidant activity are expressed as the percentage of ABTS radical inhibition. Different letters (a, b and c) indicate significant differences between samples. The same letters within the same concentration do not differ significantly. The data were analyzed via one-way ANOVA and Tukey’s post hoc test (*p* < 0.05).

**Figure 2:**
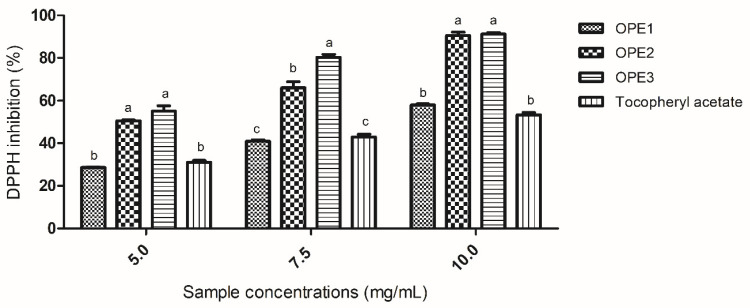
The results of the analysis of antioxidant activity are expressed as the percentage of DPPH radical inhibition. Different letters (a, b and c) indicate significant differences between samples. The same letters within the same concentration do not differ significantly. The data were analyzed via one-way ANOVA and Tukey’s post hoc test (*p* < 0.05).

The antioxidant capacity of olive pomace extracts (OPE1, OPE2, and OPE3) was evaluated via the inhibition of ABTS (Figure 1) and DPPH (Figure 2) radicals via colorimetric, indirect, and electron transfer-based methods. To evaluate the antioxidant efficacy of the samples, it is crucial to employ complementary assays to ensure a comprehensive evaluation [41]. The structure of phenolic compounds is a determining factor in their radical inhibition activity; the hydroxyls that make up phenolic compounds act as electron donors, allowing the neutralization of reactive oxygen species and the formation of radicals via a mesomeric effect [42]. The olive pomace extracts were analyzed and compared with tocopheryl acetate at different concentrations. Tocopheryl acetate was chosen because it is an antioxidant which is widely used in cosmetics and food [43].

With respect to the ABTS radical scavenging activity, both extracts with 1,3-propanediol showed greater activity against the radical than the aqueous extract. Among all the extracts analyzed, the one obtained using 1,3-propanediol alone (OPE3) presented the highest ABTS deactivation activity, even compared to tocopherol acetate, at all the concentrations tested. These comparisons highlight the potential of the extraction method employed in this study to yield olive pomace extracts with enhanced antioxidant properties. Karaosmaglu et al. [44] examined methanolic extracts of olive oil rich in phenolic compounds and reported between 1.31% and 21.97% ABTS free radical scavenging activity, demonstrating the effectiveness of the extraction method used herein in the recovery of antioxidant bioactive compounds.

The concentration of an antioxidant agent required to decrease the initial absorbance of ABTS by 50% (IC_50_) is a widely used parameter to represent the activity of antioxidants. Table 3 shows the results of this analysis and the comparison of OPE1, OPE2, OPE3, tocopheryl acetate, and BHT.

Compared to tocopherol acetate, olive pomace extracts demonstrated a greater capacity to inhibit ABTS. Among the extracts, the same trend was observed for the reduction in the Folin–Ciocalteu and total flavonoids, with an emphasis on the extracts produced with 1,3-propanediol. These results prove the potential for the application of olive pomace extracts as natural antioxidants.

The percentage of inhibition of the DPPH radical was analyzed, and the results are shown in Figure 2.

As expected, the DPPH radical inhibition capacity was concentration-dependent for all the samples tested, as were the results of the ABTS inhibition assay. However, the inhibition values were greater, demonstrating the greater effectiveness of the samples against this radical. The aqueous extract (OPE1) demonstrated a DPPH inhibition capacity similar to that of tocopheryl acetate across all the tested concentrations. Moreover, the 1,3-propanediol extracts (OPE2 and OPE3) consistently demonstrated greater DPPH radical scavenging activity than both the aqueous extract and tocopherol acetate. At a concentration of 7.5 mg/mL, OPE3 presented a greater DPPH inhibition capacity than OPE2 did, whereas at concentrations of 5 and 10 mg/mL, both extracts, OPE2 and OPE3, presented the same DPPH inhibition capacity.

Table 4 presents the DPPH radical inhibition capacity expressed as the IC_50_ values (the concentration of antioxidant required to reduce the absorbance by 50%). On the basis of these previous results, OPE3 was chosen for comparison with BHT and tocopheryl acetate. OPE3 presented an intermediate IC_50_ value, with better antioxidant activity than tocopheryl acetate. Overall, these results showed that olive pomace extracts could be used to replace tocopheryl acetate as an antioxidant in formulations.

The cosmetic and dermatological interest in phenolic compounds derived from plants is based mainly on their antioxidant capacity, and their topical application minimizes oxidative damage to the skin, in addition to providing photoprotective activity and assisting in providing therapy for the skin barrier [5,42].

### 3.3. Antioxidant Capacity of the Beta-Carotene/Linoleic Acid System

**Figure 3:**
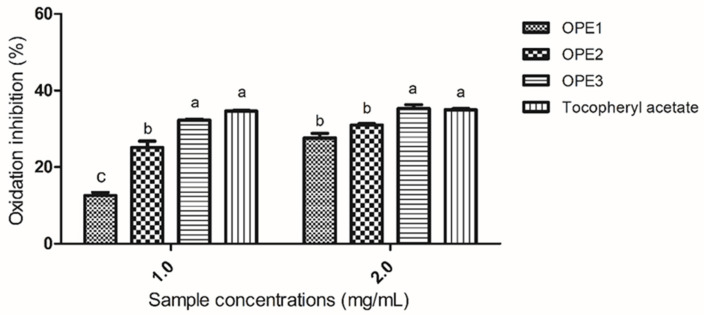
The results are expressed as the percentage of oxidation inhibition. Different letters (a, b and c) indicate significant differences between samples. The same letters within the same concentration do not differ. The data were analyzed via one-way ANOVA and Tukey’s post hoc test (*p* < 0.05).

The ability of olive pomace extracts to inhibit beta-carotene oxidation was evaluated at two different concentrations (1 and 2 mg/mL). The results are presented in Figure 3.

Compared to other olive pomace extracts, OPE3 significantly differed in its ability to inhibit oxidation and has the same antioxidant capacity as tocopheryl acetate at both concentrations tested. The ability to protect β-carotene bleaching from degradation generated by the linoleic acid oxidation products found in this study agrees with the values demonstrated in the literature, such as the findings of Karaosmaglu et al. [44], who examined methanolic extracts of olive oil rich in phenolic compounds and reported that the antioxidant capacity of the beta-carotene/linoleic acid system ranged from 21 to 64%. A commercial olive extract for topical use reduced skin lipid peroxidation by 27% after sun exposure [42].

Tocopheryl acetate was used as the reference standard in this assay because beta-carotene can inhibit singlet oxygen and interact with tocopheryl acetate to prevent lipid peroxidation, making it the appropriate control in this assay [45]. Notably, the antioxidant performance of OPE3 was comparable to that of tocopheryl acetate, highlighting its potential as a natural alternative with similar efficacy.

### 3.4. Stability Studies

**Figure 4:**
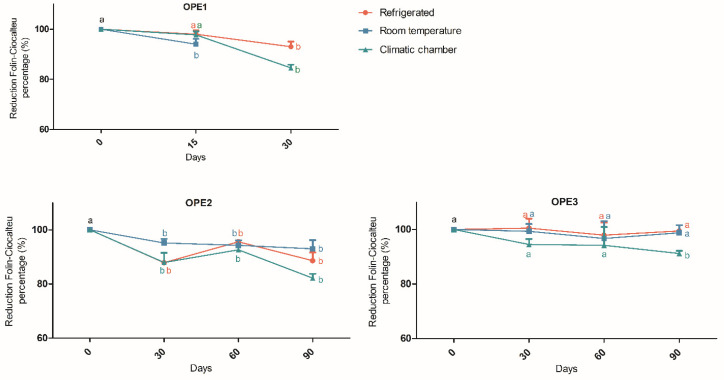
Analysis of the stability of olive pomace extracts (expressed as percentages of Folin–Ciocalteu reducing capacity). Different letters (a and b) indicate significant differences between samples. Different letters indicate significant differences. The data were analyzed via one-way ANOVA and Dunnett’s post hoc test (*p* < 0.05).

The reducing capacity of the Folin–Ciocalteu reagent was employed to assess the stability of the OPE1, OPE2, and OPE3 extracts, and the results are depicted in Figure 4.

Stability testing of the aqueous extract (OPE1) was limited to 30 days due to microbial growth, which occurred after 15 days for samples stored at room temperature. During the storage period, OPE2 showed evident variations in the analyses, especially on the 60th day, under refrigerated and climate chamber conditions. This may be explained by oxidation/reduction reactions that can modify phenolic compounds, potentially leading to the formation of new phenolic compounds or the transformation of precursors into more active compounds and other derivatives [31]. Furthermore, the Folin–Ciocalteu reduction method presents technical limitations, as the reagents used in the spectrophotometric analysis react with all the reactive hydroxyl groups present in the matrix, not exclusively with polyphenols [46]. After 90 days, OPE2 presented a maximum reduction of 18% in reducing capacity. Conversely, the OPE3 extract only significantly reduced the reducing capacity under the most severe storage conditions (climatic chamber) and after 90 days. However, this reduction was only 8%. These findings indicate that the use of 1,3-propanediol, in addition to being a more suitable extraction solvent, also contributes to the stability of the system. Indeed, previous studies have reported the antimicrobial activity of 1,3-propanediol [47].

### 3.5. Simultaneous Determination of Hydroxytyrosol and Oleuropein via HPLC-DAD

**Figure 5:**
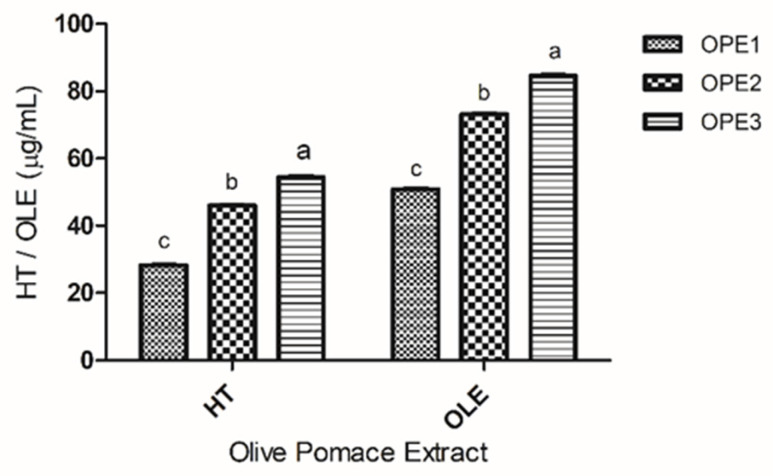
Determination of hydroxytyrosol (HT) and oleuropein (OLE) contents in olive pomace extracts. The results are presented as µg/mL in the extract. Different letters (a, b and c) in each compound analysis indicate significant differences. The data were analyzed via one-way ANOVA and Tukey’s post hoc test (*p* < 0.05).

OPE1, OPE2, and OPE3 extracts were analyzed by HPLC-DAD for the simultaneous determination of hydroxytyrosol (HT) and oleuropein (OLE) (Figure 5). Hydroxytyrosol and oleuropein are compounds generally found in olives and their derivatives, and olive pomace is an important source of hydroxytyrosol [24]. These compounds exhibit numerous beneficial biological activities, attracting interest from the cosmetic and pharmaceutical industries [43]. Both phenolic compounds were identified and quantified in the olive pomace extracts, with there being a higher concentration of oleuropein than of hydroxytyrosol in all the samples tested.

Hydroxytyrosol, a phenolic alcohol, is a product of oleuropein hydrolysis and possesses significant antioxidant properties, surpassing those of other phenolic compounds and natural antioxidants such as vitamin C, as well as synthetic antioxidants such as butylated hydroxytoluene (BHT). In the skin, it has important activities, including anti-inflammatory and photoprotective effects, and acts as an inhibitor of tyrosinase and senescence-associated β-galactosidase [24]. Hydroxytyrosol enhances the proliferation of keratinocytes, indicating its involvement in repair, regeneration, and epithelial differentiation mechanisms, as evidenced by a significant increase in the expression of Ki67 (Ki-67 antigen). Furthermore, it reduces inflammation in the human epidermis by preventing the secretion of inflammatory cytokines (IL-1 and IL-8), lowering NF-κB (nuclear factor kappa-light-chain-enhancer of activated B cells) levels, and inhibiting inducible nitric oxide synthase (iNOS) and cyclooxygenase-2 (COX-2). Hydroxytyrosol also has photoprotective effects by reducing the protein damage caused by UVA radiation and UVB-induced genotoxicity. Thus, it contributes to the maintenance of the epidermal barrier [24,42,48].

Among the biological activities of oleuropein, its antioxidant capacity and ability to chelate metal ions such as copper (Cu) and iron (Fe) stand out, preventing these ions from generating free radicals and scavenging superoxide anions [49]. Oleuropein exerts beneficial effects on the skin barrier by modulating various cellular and molecular processes. Owing to the presence of an ortho-diphenolic group in its structure, oleuropein can eliminate ROS through hydrogen donation and stabilize oxygen radicals via intramolecular hydrogen bonding [50]. In addition, oleuropein stimulates the synthesis of antioxidant enzymes via the NRF2 pathway and promotes epidermal cell proliferation while efficiently inhibiting TEWL [42]. According to Asghariazar et al. [51], this phenolic molecule influences the expression of caspases 3 and 9, promoting the regulation of apoptosis and cell renewal, which are essential for epidermal homeostasis. Furthermore, it reduces the expression of NF-κB, contributing to the attenuation of local inflammation. The regulation of peroxisome proliferator-activated receptor gamma (PPAR-γ) by oleuropein supports the maintenance of the lipid function of the skin barrier, whereas the modulation of interferon gamma (IFN-γ), a cytokine produced by resident T lymphocytes in the epidermis, assists in the mediation of cutaneous immune responses.

### 3.6. Qualitative Analysis by UHPLC-QqTOF-MS

OPE3 was analyzed via UHPLC-QqTOF-MS, and a total of 33 compounds were found via their fragment ions and isotopic profile (Table 5). The data included information on the theoretical and experimental *m*/*z* values, Milisigma (mSigma) values, retention times of the compounds, and characteristic fragment ions.

The compounds identified had mSigma values ranging from 1.8 to 34.6, corresponding to oleoside and dihydrooleuropein, respectively. The Milisigma (mSigma) value is a numerical value that indicates how similar the theoretical and measured isotopic patterns are, representing the profile of isotopic similarity. A low mSigma value indicates that the isotopic pattern found in the peak is very similar to the theoretical isotopic pattern for the proposed molecular formula. The tolerance for the mSigma value is typically set at 50 [52]. The errors associated with the analysis were consistently low for all the detected compounds, confirming their presence in the OPE3 extract. These compounds are classified into different categories and include simple phenols, iridoid precursors, secoiridoids, flavonoids, lignans, and others.

**Figure 6:**
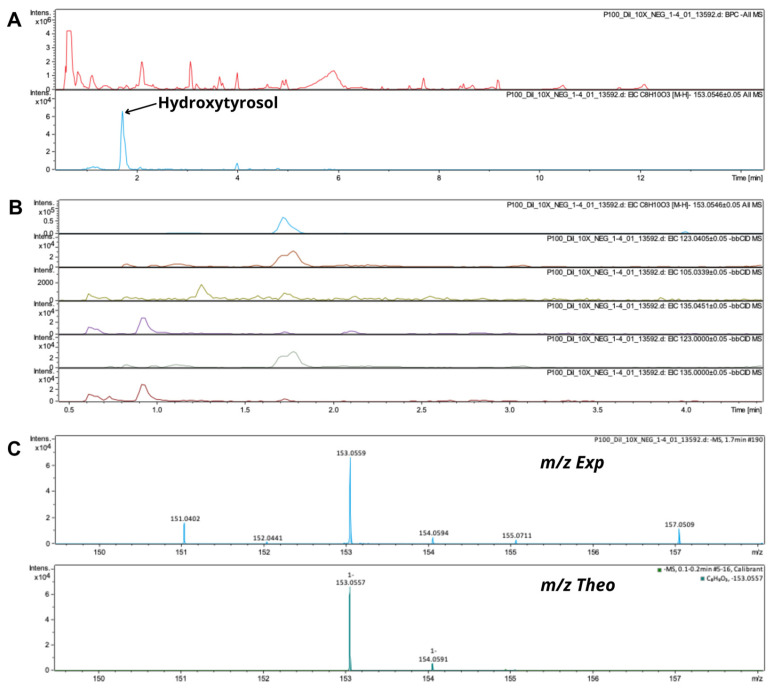
(**A**) MS/MS spectra of OPE3 and hydroxytyrosol; (**B**) hydroxytyrosol and its fragments; (**C**) theoretical and experimental isotopic profiles.

The simple phenols identified in the OPE3 extract were hydroxytyrosol, tyrosol, dihydroxytyrosol, tyrosol glucoside, and hydroxytyrosol glucoside; these compounds are responsible for the biological activities of olive plant derivatives [52]. The hydroxytyrosol glucoside generates representative fragments [53], one of which corresponds to hydroxytyrosol. The chromatographic signal attributed to hydroxytyrosol glucoside, followed by the signal of dihydroxytyrosol, was more intense than the signal of hydroxytyrosol, as reported by Peralbo-Molina et al. [33]. The mass profile of hydroxytyrosol confirmed the compatibility between the theoretical and experimental data, which was supported by the low mSigma values (Figure 6). Hydroxytyrosol was detected with a precursor ion at *m*/*z* 153.0557, which generated fragment ions at *m*/*z* 123.0405, 105.0339, and 135.0451. Klen et al. [54] reported fragment ion 123 for hydroxytyrosol. Cádiz-Gurrea et al. [22] reported fragment ions at *m*/*z* 123 and 135 for hydroxytyrosol in an olive pomace extract. D’Antuono et al. [55] reported a fragment of *m*/*z* 123 for hydroxytyrosol.

**Figure 7:**
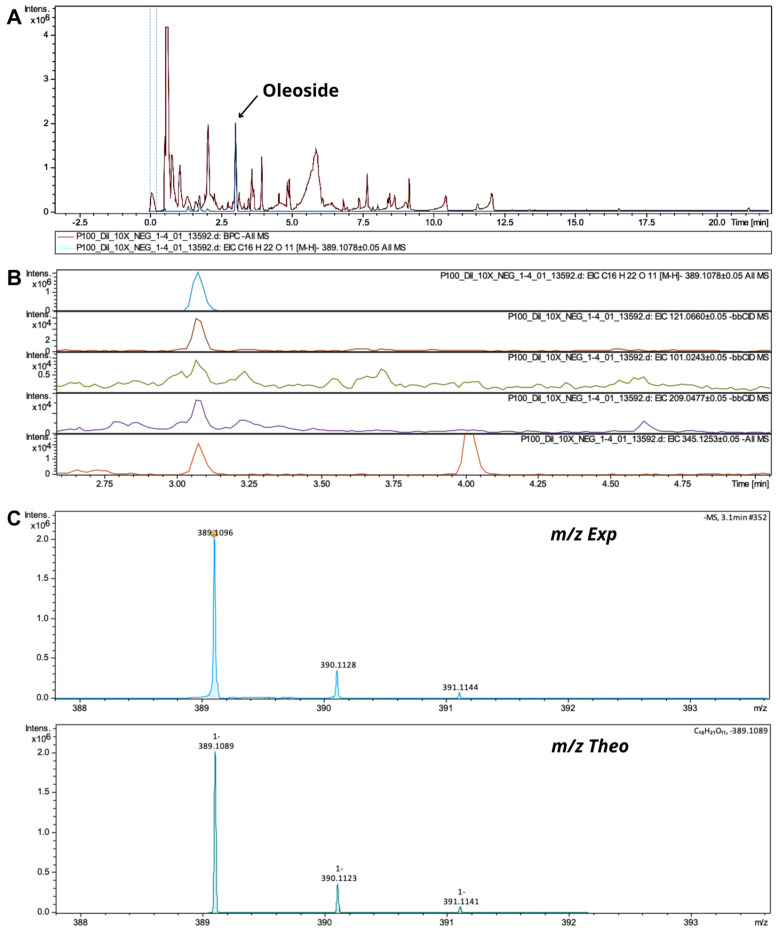
(**A**) MS/MS spectra of oleoside and OPE3; (**B**) oleoside and its fragments; (**C**) theoretical and experimental isotopic profiles.

Iridoid is a term used to designate numerous groups of monoterpenes and glycoside derivatives, the precursor of which is mevalonic acid [33]. Several iridoid precursors were presented in the OPE3 analysis; these compounds are widely described as constituents of olive extracts and their derivatives. The compounds found were loganin, secologanin, loganic acid, oleoside, oleoside dimethyl ester, oleoside glycoside, oleoside riboside, and oleoside-11-methyl ester [54]. Iridoids are bioactive compounds widely used to treat skin disorders because of their anti-inflammatory and antioxidant properties [22]. Oleoside was found with a precursor ion at *m*/*z* 389.1089 and fragment ions at *m*/*z* 121.0660, 101.0243, 209.0477, and 345.1253. Klen et al. [54], when analyzing an olive byproduct extract, reported fragments at *m*/*z* 345 and 209 for oleoside, and Serrano-Garcia et al. [53] reported a fragment at *m*/*z* 345.1180 (Figure 7).

**Figure 8:**
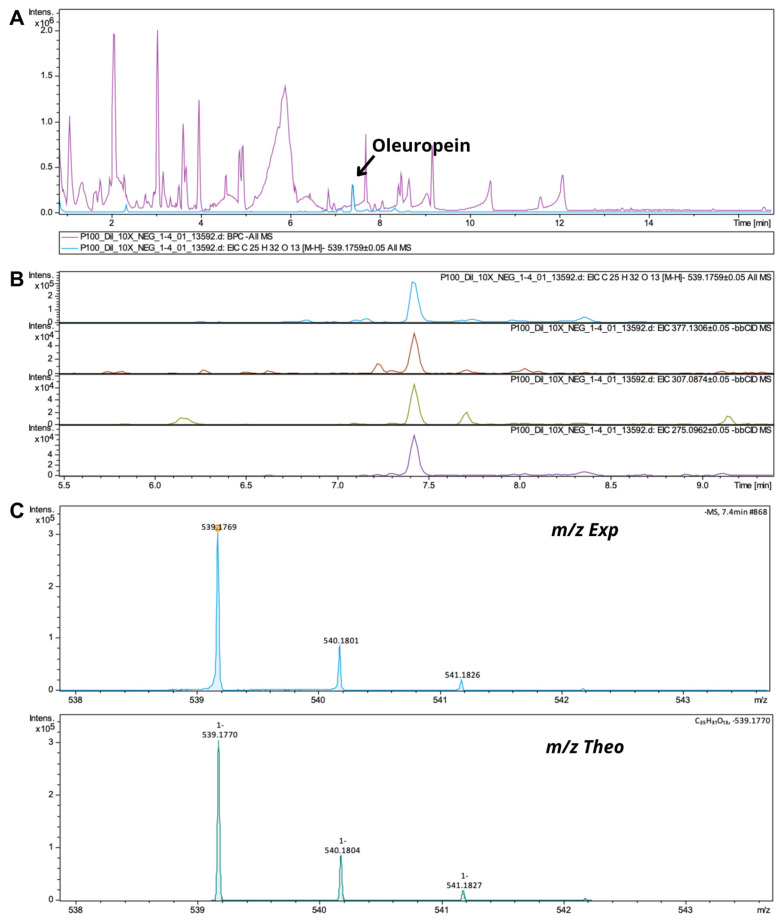
(**A**) MS/MS spectra of oleuropein and OPE3; (**B**) oleuropein and its fragments; (**C**) theoretical and experimental isotopic profiles.

Phenolics classified as secoiridoids are characterized by the presence of the antioxidant oleic acid or its derivatives [33]. Secoiridoids are phenolic compounds unique to Oleaceae plants [56]. Several secoiridoids, including comselogoside, secologanoside, caffeoyl-6′-secologanoside, oleuropein, ligstroside (p-HPEA-EA), and verbascoside, have been identified. Oleuropein derivatives, such as decarboxymethylated glycones, known as oleacein or 3,4-DHPEA-EDA; oleuropein aglycones [57]; dihydrooleuropein [58]; oleuropein derivative 2; and 10-hydroxyoleuropein [33], were also detected in OPE3. Oleuropein was detected with a precursor ion at *m*/*z* 539.1770. The fragmentation of this ion resulted in fragments with *m*/*z* 377.1306 due to hexose unit cleavage, and 307.0874 and 275.0962, characteristic of the oleuropein structure (Figure 8).

The flavonoids identified in OPE3 include luteolin, rutin, taxifolin, luteolin glucoside, and apigenin glucoside. Rutin was detected at *m*/*z* 609.1461, which displayed a fragment ion at *m*/*z* 300.0274 resulting from the loss of the glycoside and rhamnoside groups, along with another fragment at *m*/*z* 197.0000. Luteolin glucoside, detected at 6.2 min with *m*/*z* 447.0933, yielded a fragment ion at *m*/*z* 285.0423 due to the loss of a hexose unit. Taxifolin, also known as dihydroquercetin, was also detected, which agreed with the findings of Cádiz-Gurrea et al. [22].

Other simple phenols, such as vanillin or vanillic acid, which are isomers of coumaric acid, have also been found in OPE3 extracts. Similarly, benzyl primeveroside was identified as a precursor of the aroma, as reported by Cádiz-Gurrea et al. [22]. Lignans such as pinoresinol and acetoxypinoresinol, which are recognized for their antioxidant and antitumor properties, have also been found [59,60].

Olive pomace extracts offer a variety of interesting cosmetic bioactives and, consequently, synergistic effects, as interactions among phenolics or between phenolics and other antioxidants can increase their activity. For example, a study by Cádiz-Gurrea et al. [22], who analyzed polyphenol-rich extracts from olive oil byproducts for cosmetic applications, identified numerous phenolic compounds, indicating diverse and multifunctional compositions. The synergistic activity of these compounds is considered responsible for their beneficial effects on the skin, including their antioxidant and anti-inflammatory actions; the inhibition of degenerative enzymes; their photoprotective activity; the reduction in melanin biosynthesis; their antiaging properties; and their therapeutic potential for conditions such as psoriasis, allergic dermatitis, seborrhea, and microbial infections. Carrara et al. [42] conducted a study using hydroxytyrosol- and oleuropein-rich extracts, which demonstrated accelerated cell restoration, increased levels of growth factors, and a two-fold increase in epithelial cell proliferation. The study also revealed that the individual compounds were less effective when tested separately, suggesting that phenolic compounds exhibit greater activity when administered as a complex extract rather than in their purified form.

Moreover, the use of natural and safe solvents, such as 1,3-propanediol, aligns with the principles of green chemistry and contributes to both the effectiveness and overall sustainability of cosmetic products.

Further information on the phenolic composition of OPE3 is available in the Supplementary Material.

### 3.7. Antiglycation Activity

Glycation is a nonenzymatic reaction in which reducing sugars bind to primary or secondary amino groups, leading to the formation of advanced glycation end products [61]. This process, involving free radicals and carbonyl intermediates, occurs naturally during aging and is related to various pathological processes [62]. The antiglycation activity of OPE3 was analyzed at two different concentrations (2 and 3.5 mg/mL). As shown in Table 6, the antiglycation effect was dose dependent and may have been related to the presence of phenolic compounds in the extract.

For comparison, a positive control containing carnosine (0.3 mg/mL), a well-known antiglycation compound, was selected and tested at a concentration recommended for cosmetic use. Ramkissoon et al. [63] confirmed the correlation between the antioxidant activity, total phenolic content, and antiglycation capacity of medicinal herb extracts. Márquez et al. [61] developed an olive leaf extract with strong antiglycation effects. Kabach et al. [64] reported significant antiglycation activity in methanolic olive extracts, with 60.2% inhibition of advanced glycation end products at a concentration of 60 µg/mL. The accumulation of AGEs in the skin is a remarkable cause of aging: manifestations of AGE-induced skin aging include wrinkles, skin discoloration, reduced radiance, and impaired skin barrier function. In this way, avoiding AGEs can contribute to the health of the skin barrier [65].

### 3.8. Antioxidant Capacity of Cosmetic Formulations

**Figure 9:**
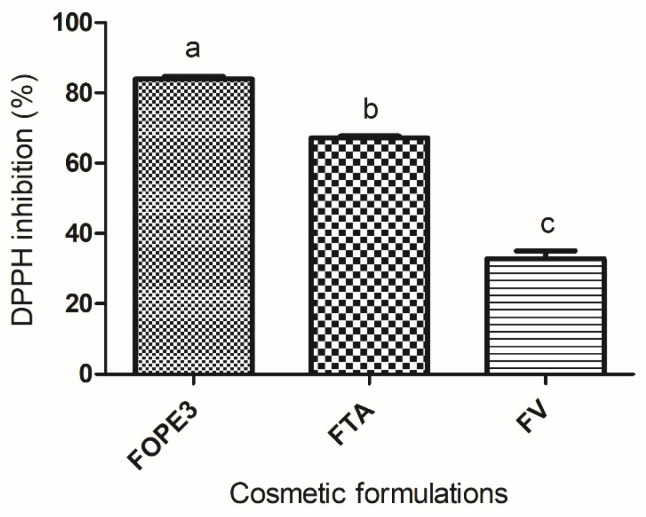
The results of the analysis of antioxidant activity are expressed as the percentage of DPPH radical inhibition. FOPE3: cosmetic formulation containing OPE3; FTA: cosmetic formulation containing tocopheryl acetate; FV: vehicle formulation. Different letters (a, b and c) indicate significant differences. The data were analyzed via one-way ANOVA and Tukey’s post hoc test (*p* < 0.05).

Antioxidants are suitable ingredients for use in cosmetic formulations, not only because of their ability to inhibit and stabilize free radicals but also because of their potential to protect against photodamage and antiaging effects [12]. Cosmetic formulations containing OPE (FOPE3), tocopheryl acetate, and vehicle were analyzed for their antioxidant potential via the DPPH radical inhibition method (Figure 9).

The formulation containing OPE3 (FOPE3) demonstrated the highest degree of radical inhibition, significantly surpassing that of tocopheryl acetate and the vehicle. Considering that tocopheryl acetate is a widely used antioxidant in the cosmetic industry [66], these results confirm the potential of olive pomace extract as an antioxidant in cosmetic formulations.

## 4. Conclusions

This study successfully developed a sustainable and efficient extraction process for olive pomace, utilizing simple techniques and the natural solvent 1,3-propanediol. This solvent demonstrated superior performance in extracting phenolic compounds, resulting in an extract rich in these valuable phytochemicals. A total of 33 phenolic compounds were identified, with oleuropein and hydroxytyrosol being particularly prominent.

The resulting extracts exhibited remarkable in vitro antioxidant capacity, effectively inhibited ABTS and DPPH radicals, and demonstrated protective effects within the beta-carotene/linoleic acid system. Furthermore, the extract obtained with 1,3-propanediol maintained its reducing capacity for 90 days and displayed significant antiglycation activity. Notably, its antioxidant capacity surpassed that of tocopheryl acetate, both in its pure form and when incorporated into a cosmetic emulsion. This superior performance suggests its strong potential as a natural ingredient for cosmetic applications. Given that oxidative stress directly compromises skin barrier function, this enhanced antioxidant capacity implies that the extract has the potential to indirectly contribute to skin barrier homeostasis by mitigating oxidative damage to its structural components.

In summary, this research highlights the sustainable and viable repurposing of olive pomace as a renewable source of natural raw material for the cosmetics sector. The high antioxidant capacity of the obtained extract makes it a promising alternative to widely used cosmetic ingredients such as tocopherol acetate. The implementation of effective extraction techniques, combined with the use of a natural and nontoxic solvent, ensures both the sustainability of the production process and the safety of the resulting ingredients. Moreover, the favorable physicochemical properties of the extracts facilitate their direct integration into topical formulations, thereby accelerating their technological transfer for commercial applications. Furthermore, the efficiency of the extraction process and the high availability of olive pomace as a raw material support the feasibility of scaling up this method for industrial production. These qualities establish the extract as a promising candidate for commercial development in the cosmetics industry.

The forthcoming research will advance the practical application of olive pomace extract by thoroughly assessing its safety, including its cytotoxicity and biological performance. These steps are crucial for ensuring the successful and safe application of the extracts in skincare products. Subsequently, clinical trials are essential to confirm the extract biocompatibility and in vivo efficacy of the extract.

## 5. Patents

The process of extracting antioxidant bioactive compounds from olive pomace has a patent application for invention with the National Institute for Intellectual Protection (INPI) under process number BR 20240036697.

## Figures and Tables

**Table 1:** Composition of the cosmetic emulsion formulation.

Component	Concentration (%)	Function
Potassium cetyl phosphate	0.5	emulsifier, surfactant
Cetearyl olivate (and) sorbitan olivate	1.0	emulsifier, stabilizer
Caprylic/Capric triglycerides	5.0	emollient
*Lonicera japonica* flower extract and *Lonicera caprifolium* flower extract	1.0	preservative
Ultrapure water	92.5	vehicle

**Table 2:** Characterization of OPE: reducing capacity of the Folin–Ciocalteu reagent and total flavonoid content.

Extracts	Reducing Capacity (mg GAE/g)	Total Flavonoid (mg QE/g)
OPE1	14.9 ± 0.5 ^b^	4.0 ± 0.7 ^c^
OPE2	29.0 ± 1.3 ^a^	5.0 ± 0.2 ^b^
OPE3	33.0 ± 5.7 ^a^	5.3 ± 0.2 ^a^

The values are expressed as the means ± standard deviations. Different letters (a, b and c) indicate significant differences between samples. The same letters within a column indicate the absence of a significant difference. The data were analyzed via one-way ANOVA and Tukey’s post hoc test (*p* < 0.05).

**Table 3:** ABTS radical inhibition capacity.

Antioxidant	IC_50_ (mg/mL)
Butyl hydroxytoluene (BHT)	0.024 ± 0.02 ^e^
Tocopheryl acetate	183 ± 0.6 ^a^
OPE1	19 ± 0.02 ^b^
OPE2	16 ± 0.02 ^c^
OPE3	11 ± 0.01 ^d^

The results are expressed as the IC_50_, which was calculated as the ability to reduce the sample absorbance by 50%. Different letters (a, b, c, d, and e) indicate significant differences between samples. The same letters do not differ significantly. The data were analyzed via one-way ANOVA and Tukey’s post hoc test (*p* < 0.05).

**Table 4:** DPPH radical inhibition capacity.

Antioxidant	IC_50_ (mg/mL)
Butyl hydroxytoluene (BHT)	0.1 ± 0.00 ^c^
Tocopheryl acetate	49.1 ± 1.04 ^a^
OPE3	4.4 ± 0.08 ^b^

The results are expressed as the IC_50_, which was calculated as the ability to reduce the sample absorbance by 50%. Different letters (a, b and c) indicate significant differences between samples. The same letters do not differ significantly. The data were analyzed via one-way ANOVA and Tukey’s post hoc test (*p* < 0.05).

**Table 5:** Compounds in OPE3 identified by UHPLC-QqTOF-MS.

Compounds (Molecular Formula)	Error	mSigma *	T_R_	*m*/*z* theo	*m*/*z* exp	Fragments
Hydroxytyrosol C_8_H_10_O_3_	−1.4	5.6	1.7	153.0559	153.0557	123.0405 105.0339 135.0451 123.0000 135.0000
Tyrosol C_8_H_10_O_2_	2.2	n.a.	5.6	137.0611	137.0608	119.0505 137.0603
Dihydroxytyrosol C_14_H_20_O_8_	0.3	6.9	0.9	315.1086	315.1085	151.0397 123.0448 151.0398
Tyrosol glucoside C_14_H_20_O_7_	−0.3	15.0	2.3	299.1135	299.1136	119.0505 299.1139 119.0349 101.0244 089.0245
Hydroxytyrosol glucoside C_14_H_20_O_8_	−0.3	2.1	1.8	315.1085	315.1085	315.1074 123.0446 153.0570
Loganin C_17_H_26_O_10_	−0.4	20.8	2.1	389.1452	389.1453	151.0776 113.0348 101.0206 228.0902
Secologanin C_17_H_24_O_10_	0.0	19.2	2.8	387.1297	387.1297	147.0443
Loganic acid C_16_H_24_O_10_	0.6	7.8	2.0	375.1299	375.1297	107.0506 101.0245 213.0000 113.0248
Secologanoside C_16_H_22_O_11_	−0.1	5.6	5.5	555.1719	555.1719	059.0139 089.0234
Comselogoside C_25_H_28_O_13_	0.5	3.3	7.7	535.1460	535.1457	491.1558 345.1197 145.0296 535.0000
Caffeoyl-6′-secologanoside C_25_H_28_O_14_	−0.8	10.5	6.9	551.1411	551.1406	507.1504 345.1193 281.0673 161.0245
Oleoside C_16_H_22_O_11_	−1.1	1.8	3.1	389.1094	389.1089	121.0660 101.0243 209.0477 345.1253
Oleoside dimethylester C_18_H_26_O_11_	0.1	4.5	3.2	417.1402	417.1402	185.0000 101.0000 244.0000
Oleoside glucoside C_22_H_32_O_16_	−0.2	31.6	3.2	551.1617	551.1618	551.1707 507.1805 209.0480
Oleoside riboside C_20_H_26_O_15_	0.8	27.8	4.0	505.1203	505.1199	389.1166 505.1298 345.1256
Oleoside dimethylester C_18_H_26_O_11_	0.1	4.5	3.2	417.1402	417.1402	185.0000 101.0000 244.0000
Luteolin C_21_H_20_O_10_	−1.1	23.5	6.9	431.0989	431.0984	431.0973 255.0295 284.0276
Luteolin glucoside C_21_H_20_O_11_	0.1	5.3	6.2	447.0933	447.0933	285.0423
Taxifolin C_15_H_12_O_7_	−0.7	15.9	6.6	303.0512	303.0510	179.0000 255.1114
Rutin C_27_H_30_O_16_	−0.2	7.6	6.0	609.1462	609.1461	300.0274 497.0000
Vanillin C_8_H_8_O_3_	1.6	5.6	1.8	151.0403	151.0401	151.0397 105.0341 135.9000
Benzyl b-primeveroside C18H26O10	0.0	20.3	3.5	401.1453	401.1453	223.0000
Oleuropein C_25_H_32_O_13_	0.0	6.2	7.4	539.1770	539.1770	539.1759 377.1306 307.0874 275.0962
Oleuropein aglycone C_19_H_22_O_8_	−0.2	2.4	10.5	377.1243	377.1242	111.0088 149.0244 195.0645 275.0913 307.0823
Luteolin glucoside C_21_H_20_O_11_	0.1	5.3	6.2	447.0933	447.0933	285.0423
Dihydro-oleuropein C_25_H_36_O_13_	−0.5	34.6	6.3	543.2086	543.2083	377.1522
Oleuropein diglucoside C_25_H_36_O_12_	0.0	8.3	7.2	527.2134	527.2134	377.1481
10-hydroxy-oleuropein C_25_H_32_O_14_	−0.1	5.6	5.5	555.1719	555.1719	537.1647 376.1114
Oleacein C_17_H_20_O_6_	0.6	17.4	8.5	319.1187	319.1187	139.0602 165.0556 183.0660
Acetoxypinoresinol C_22_H_24_O_8_	−0.8	23.5	9.2	415.1395	415.1398	280.0951 343.1188
Apigenin glucoside C_21_H_20_O_10_	1.1	23.5	6.9	431.0989	431.0984	269.0446 240.0462
p-HPEA-EA C_19_H_22_O_7_	−0.2	2.4	12.1	361.1294	361.1293	361.0000 139.0000
Pinoresinol C_20_H_22_O_6_	0.9	17.9	7.8	357.1347	357.1344	311.1363 175.0787 85.9461 214.3294
Verbascoside C_29_H_36_O_15_	−0.8	7.7	6.1	623.1986	623.1981	461.1744 161.0254 161.0000

* mSigma values < 50 indicate a high probability of a correct formula. T_R_ = retention time.

**Table 6:** Antiglycation capacity.

Extracts	Concentration (mg/mL)	Antiglycation Capacity (%)
OPE3	2.0	24.4 ± 3.2 ^c^
OPE3	3.5	40.4 ± 3.1 ^b^
Carnosine	0.3	91.0 ± 3.4 ^a^

The values are expressed as the means ± standard deviations. Different letters within a column indicate different results. The data were analyzed via one-way ANOVA and Tukey’s post hoc test (*p* < 0.05).

## Data Availability

The data supporting the findings of this study are presented in the article and in the Supplementary Materials. Additional information can be requested by contacting the researcher Roberta Riéffel at the e-mail address: roberta.rieffel@ufrgs.br.

## Supplementary Materials

The following supporting information can be downloaded at https://www.mdpi.com/article/10.3390/pharmaceutics17080962/s1, Figure S1: (A) MS/MS spectra of hydroxytyrosol glucoside and its fragment ions. (B) Theoretical and experimental isotopic profiles, Figure S2: (A) MS/MS spectra of tyrosol and (B) its fragment ions; (C) theoretical and experimental isotopic profiles, Figure S3: (A) MS/MS spectra of tyrosol glucoside and its fragment ions. (B) Theoretical and experimental isotopic profiles, Figure S4: (A) MS/MS spectra of oleoside 11-methylester and its fragment ions. (B) Theoretical and experimental isotopic profiles, Figure S5: (A) MS/MS spectra of the oleoside dimethylester and its fragment ions. (B) Theoretical and experimental isotopic profiles, Figure S6: (A) MS/MS spectra of oleoside glucoside and its fragment ions. (B) Theoretical and experimental isotopic profiles, Figure S7: (A) MS/MS spectra of oleoside riboside and its fragment ions. (B) Theoretical and experimental isotopic profiles, Figure S8: (A) MS/MS spectra of loganin and its fragment ions. (B) Theoretical and experimental isotopic profiles, Figure S9: (A) MS/MS spectra of loganic acid and its fragment ions. (B) Theoretical and experimental isotopic profiles, Figure S10: (A) MS/MS spectra of secologanin and its fragment ions. (B) Theoretical and experimental isotopic profiles, Figure S11: (A) MS/MS spectra of comselogoside and its fragment ions. (B) Theoretical and experimental isotopic profiles, Figure S12: (A) MS/MS spectra of dihydroxityrosol and its fragment ions. (B) Theoretical and experimental isotopic profiles, Figure S13: (A) MS/MS spectra of secologonoside and its fragment ions. (B) Theoretical and experimental isotopic profiles, Figure S14: (A) MS/MS spectra of caffeoyl-6′-secologanoside and its fragment ions; (B) theoretical and experimental isotopic profile, Figure S15: (A) MS/MS spectra of oleuropein aglycone and its fragment ions. (B) Theoretical and experimental isotopic profiles, Figure S16: (A) MS/MS spectra of dihydrooleuropein and its fragment ions. (B) Theoretical and experimental isotopic profiles, Figure S17: (A) MS/MS spectra of the oleuropein derivative and its fragment ions. (B) Theoretical and experimental isotopic profiles, Figure S18: (A) MS/MS spectra of 10-hydroxy-oleuropein and its fragment ions. (B) Theoretical and experimental isotopic profiles, Figure S19: (A) MS/MS spectra of p-HPEA-EA and its fragment ions. (B) Theoretical and experimental isotopic profiles, Figure S20: (A) MS/MS spectra of oleacein and its fragment ions. (B) Theoretical and experimental isotopic profiles, Figure S21: (A) MS/MS spectra of verbascoside and its fragment ions. (B) Theoretical and experimental isotopic profiles, Figure S22: (A) MS/MS spectra of luteolin and its fragment ions. (B) Theoretical and experimental isotopic profiles, Figure S23: (A) MS/MS spectra of luteolin glucoside and its fragment ions. (B) Theoretical and experimental isotopic profiles, Figure S24: (A) MS/MS spectra of rutin and its fragment ions. (B) Theoretical and experimental isotopic profiles, Figure S25: (A) MS/MS spectra of apigenin glucoside and its fragment ions. (B) Theoretical and experimental isotopic profiles, Figure S26: (A) MS/MS spectra of taxifolin and its fragment ions. (B) Theoretical and experimental isotopic profiles, Figure S27: (A) MS/MS spectra of vanillin and its fragment ions. (B) Theoretical and experimental isotopic profiles, Figure S28: (A) MS/MS spectra of pinoresinol and its fragment ions. (B) Theoretical and experimental isotopic profiles, Figure S29: (A) MS/MS spectra of acetoxypinoresinol and its fragment ions. (B) Theoretical and experimental isotopic profiles, Figure S30: (A) MS/MS spectra of benzyl primaveroside and its fragment ions. (B) Theoretical and experimental isotopic profiles.

## Abbreviations

The following abbreviations are used in this manuscript:

SC	Stratum corneum
ROS	Reactive oxygen species
TEWL	Transepidermal water loss
OPE	Olive pomace extract
OLE	Oleuropein
HT	Hydroxytyrosol
iNOS	Inducible nitric oxide synthase
COX-2	Cyclooxygenase-2
Ki-67	Ki-67 antigen
NF-κB	Nuclear factor kappa-light-chain-enhancer of activated B cells
Nrf2	Nuclear factor erythroid 2-related factor 2
PPAR- γ	Peroxisome proliferator-activated receptor gamma
IFN-γ	Interferon gamma
FTA FOPE3 FV	Cosmetic formulation containing tocopheryl acetate Cosmetic formulation containing OPE3 Cosmetic formulation vehicle
