# Supplementary material for: Sustainable Olive Pomace Extracts for Skin Barrier Support
Source: Pharmaceutics. 2025 Jul 25;17(8):962. doi: 10.3390/pharmaceutics17080962 (PMC12389349; doi:10.3390/pharmaceutics17080962)
Supplement: Supplementary file 1 [file pharmaceutics-17-00962-s001.zip › pharmaceutics-3748696-supplementary.pdf]

SUPLEMENTARY MATERIAL

ANALYSIS OF THE PHENOLIC COMPOSITION AND ANTIOXIDANT ACTIVITY OF SUSTAINABLE EXTRACTS OBTAINED FROM OLIVE POMACE

FIGURES: *Qualitative analysis by UHPLC-QqTOF-MS of OPE3*

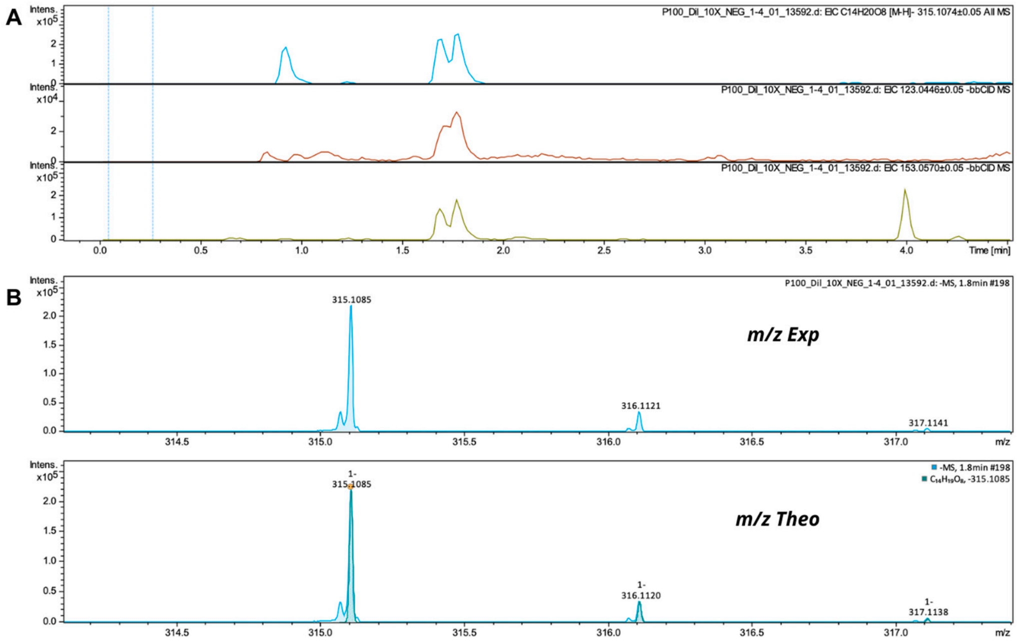

Figure S1. (A) MS/MS spectrum for hydroxytyrosol glucoside and its fragment ions; (B) Theoretical and experimental isotopic profile.

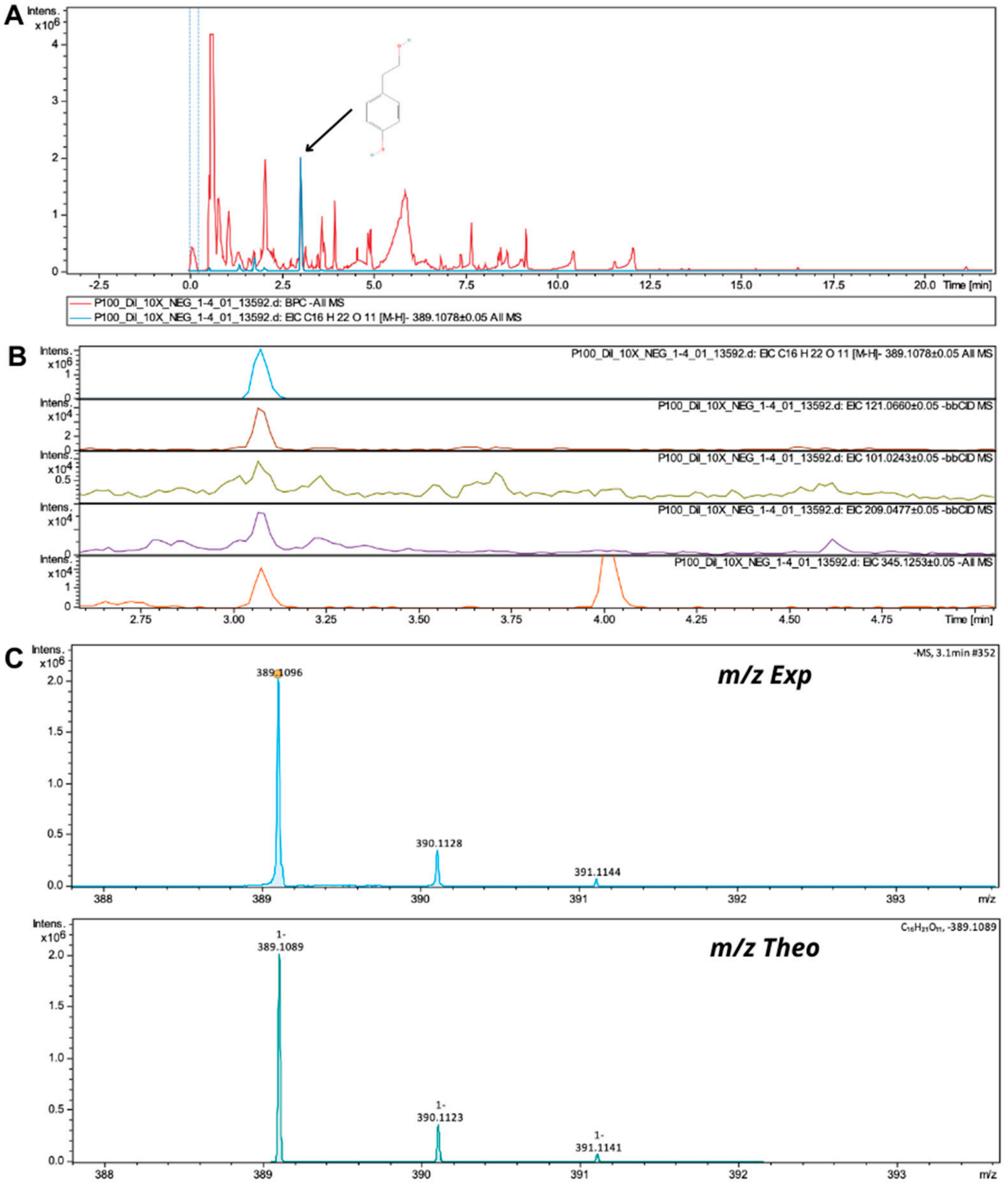

Figure S2. (A) MS/MS spectrum for tyrosol and (B) its fragment ions; (C) Theoretical and experimental isotopic profile.

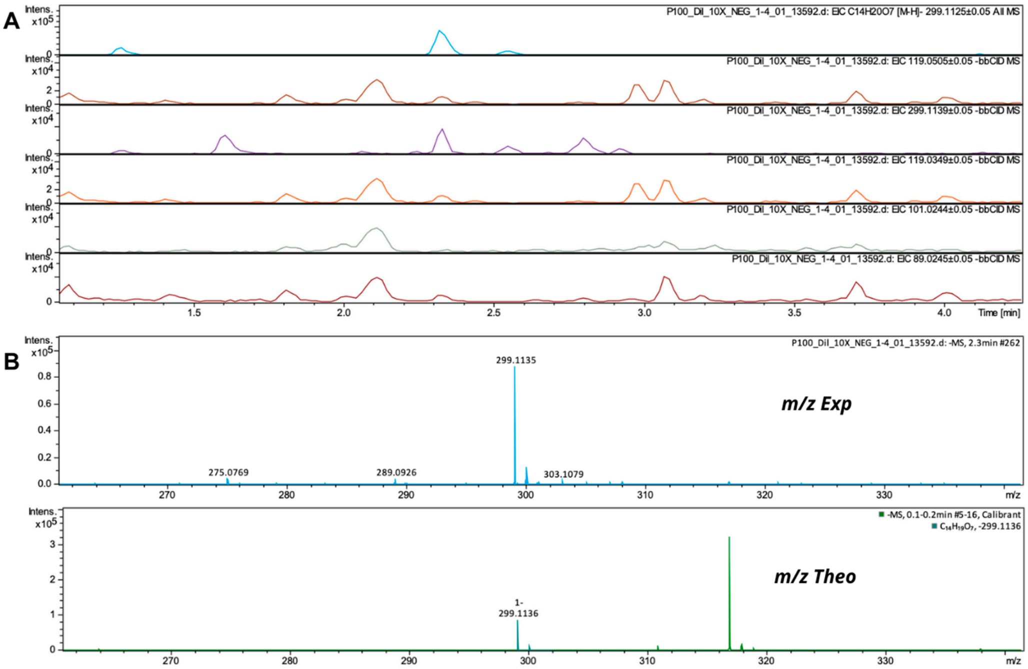

Figure S3. (A) MS/MS spectrum for tyrosol glucoside and its fragment ions; (B) Theoretical and experimental isotopic profile.

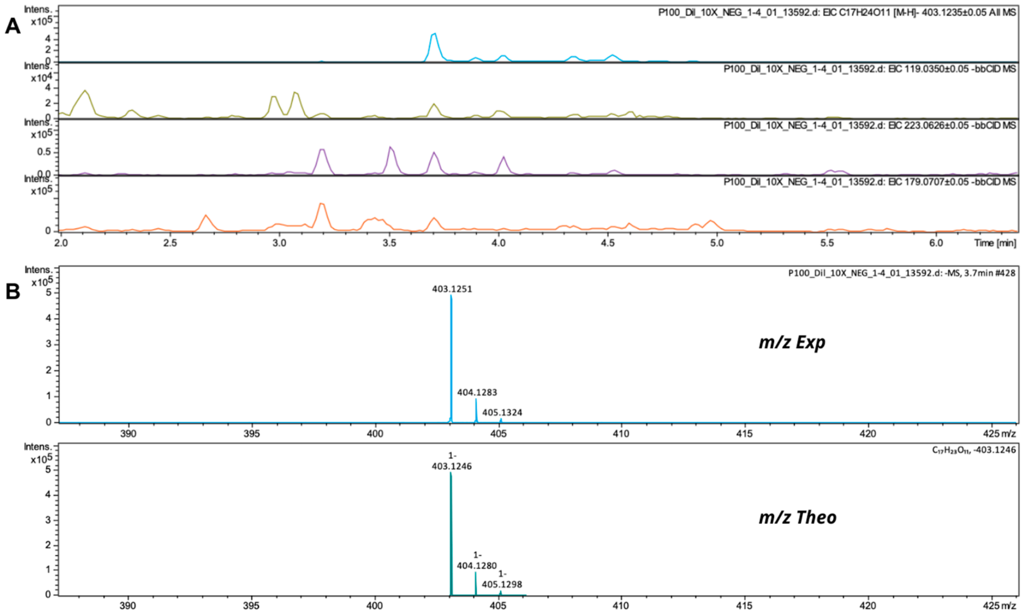

Figure S4. (A) MS/MS spectrum for oleoside 11-methylester and its fragment ions; (B) Theoretical and experimental isotopic profile.

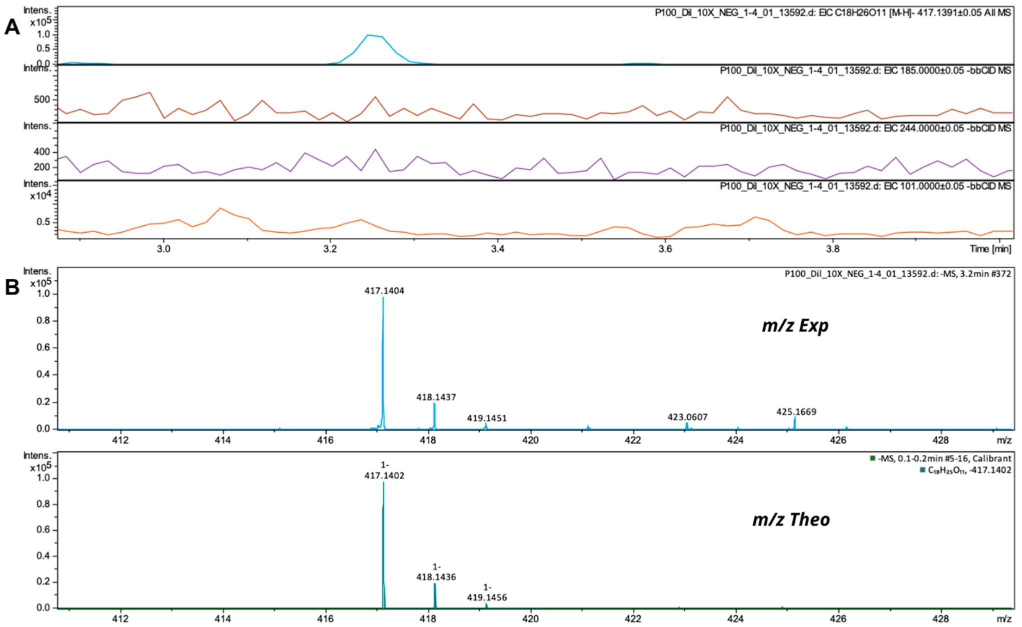

Figure S5. (A) MS/MS spectrum for oleoside dimethylester and its fragment ions; (B) Theoretical and experimental isotopic profile.

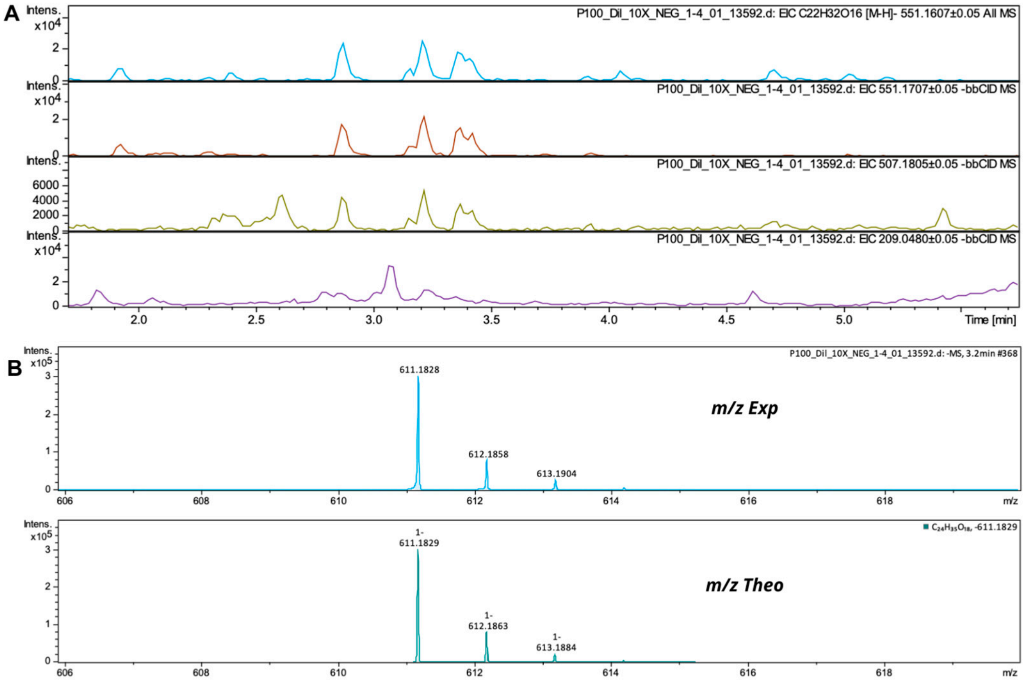

Figure S6. (A) MS/MS spectrum for oleoside glucoside and its fragment ions; (B) Theoretical and experimental isotopic profile.

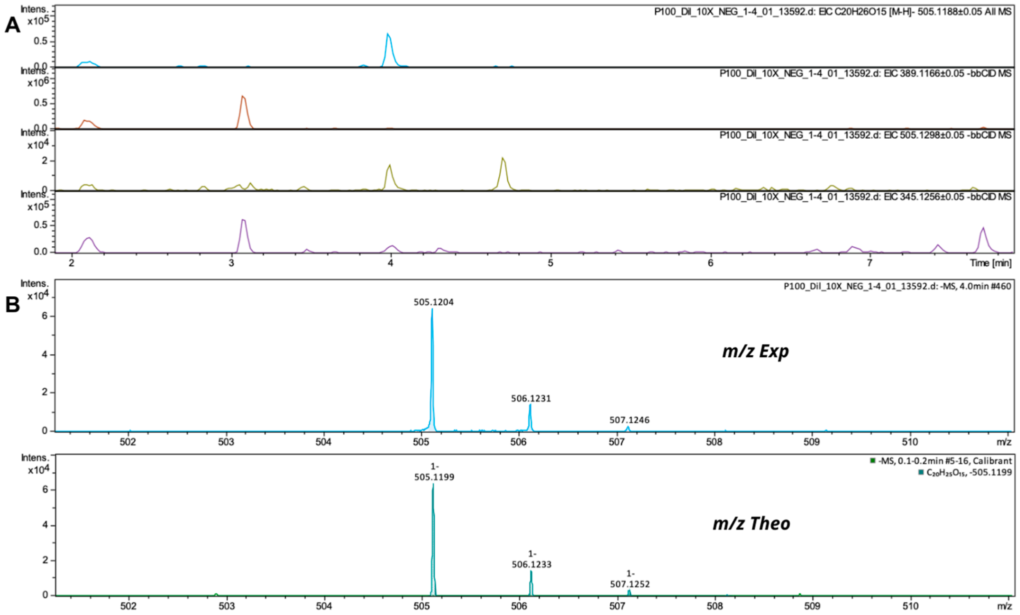

Figure S7. (A) MS/MS spectrum for oleoside riboside and its fragment ions; (B) Theoretical and experimental isotopic profile.

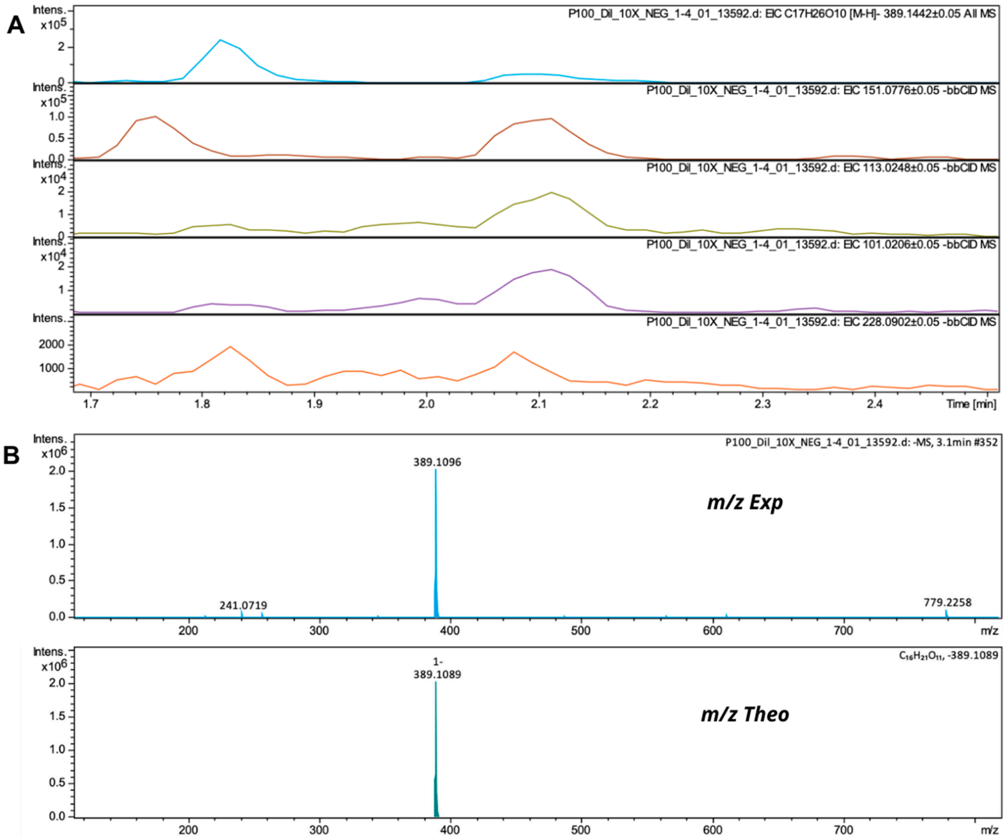

Figure S8. (A) MS/MS spectrum for loganin and its fragment ions; (B) Theoretical and experimental isotopic profile.

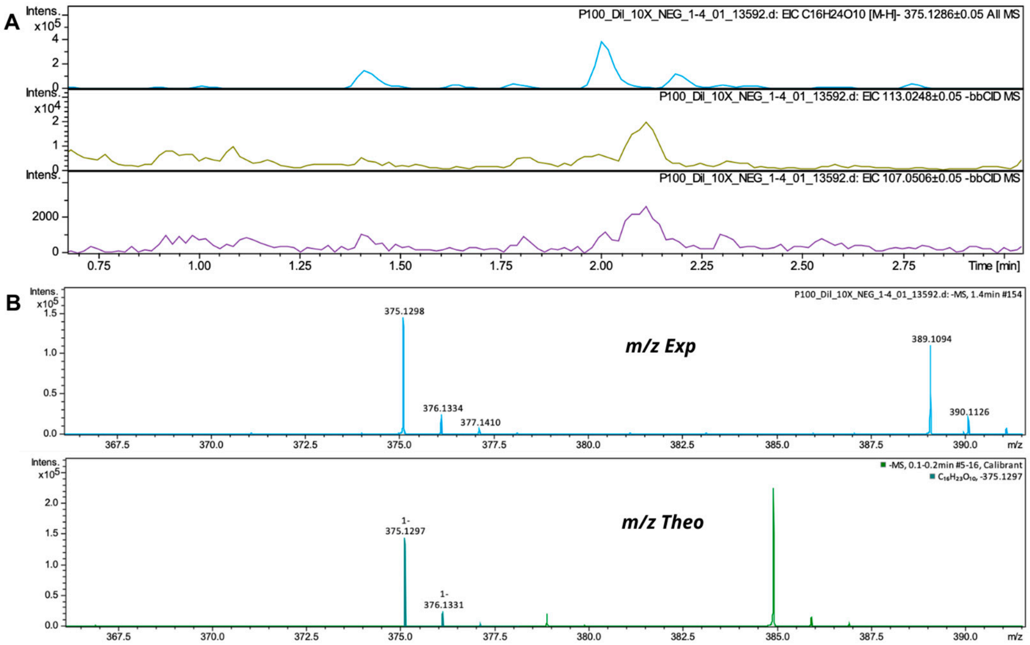

Figure S9. (A) MS/MS spectrum for loganic acid and its fragment ions; (B) Theoretical and experimental isotopic profile.

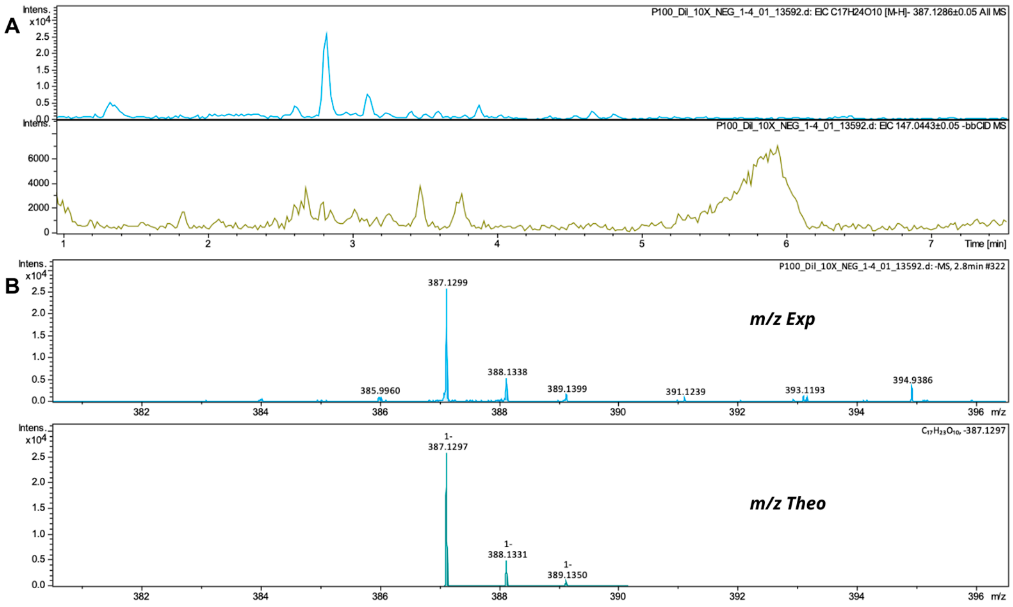

Figure S10. (A) MS/MS spectrum for secologanin and its fragment ions; (B) Theoretical and experimental isotopic profile.

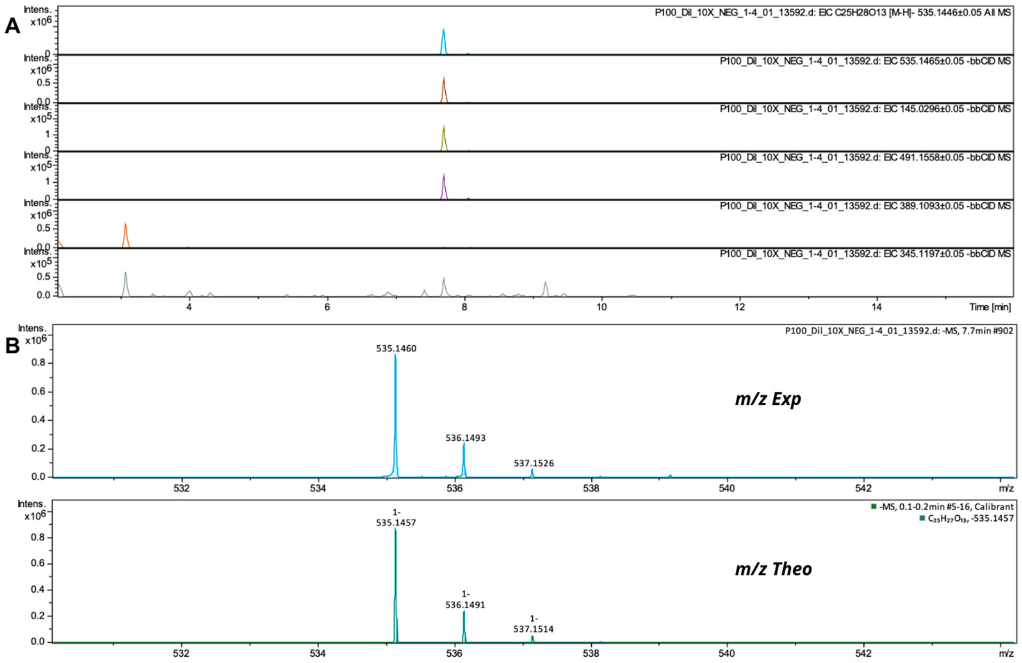

Figure S11. (A) MS/MS spectrum for comselogoside and its fragment ions; (B) Theoretical and experimental isotopic profile.

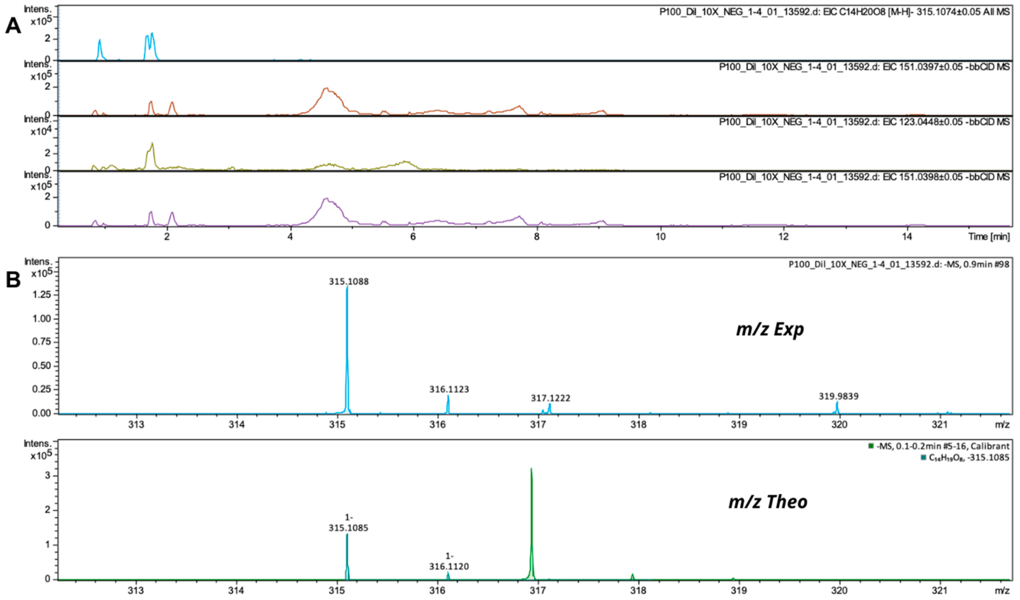

Figure S12. (A) MS/MS spectrum for dihydroxityrosol and its fragment ions; (B) Theoretical and experimental isotopic profile.

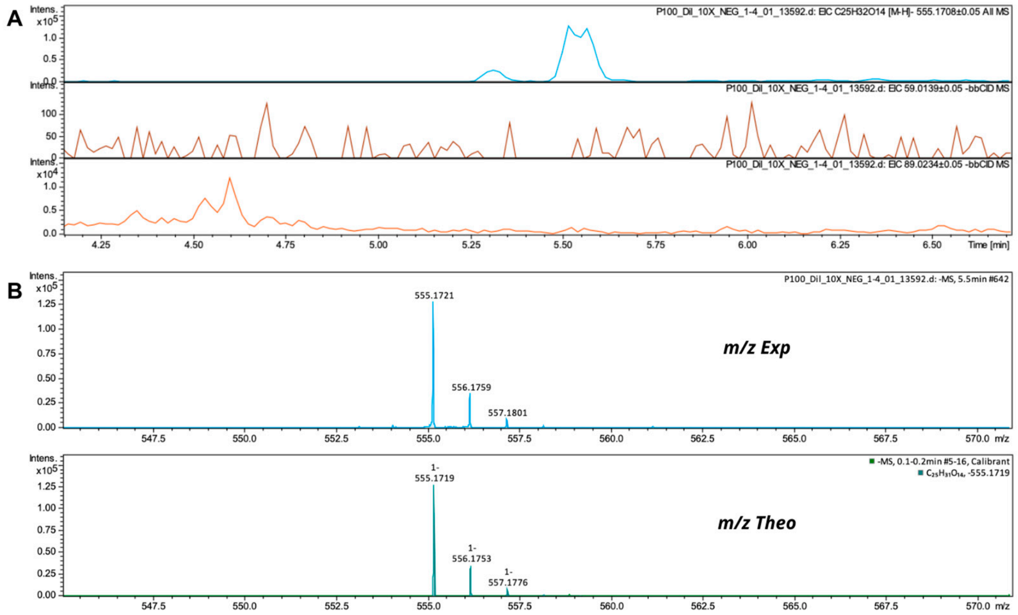

Figure S13. (A) MS/MS spectrum for secologonoside and its fragment ions; (B) Theoretical and experimental isotopic profile.

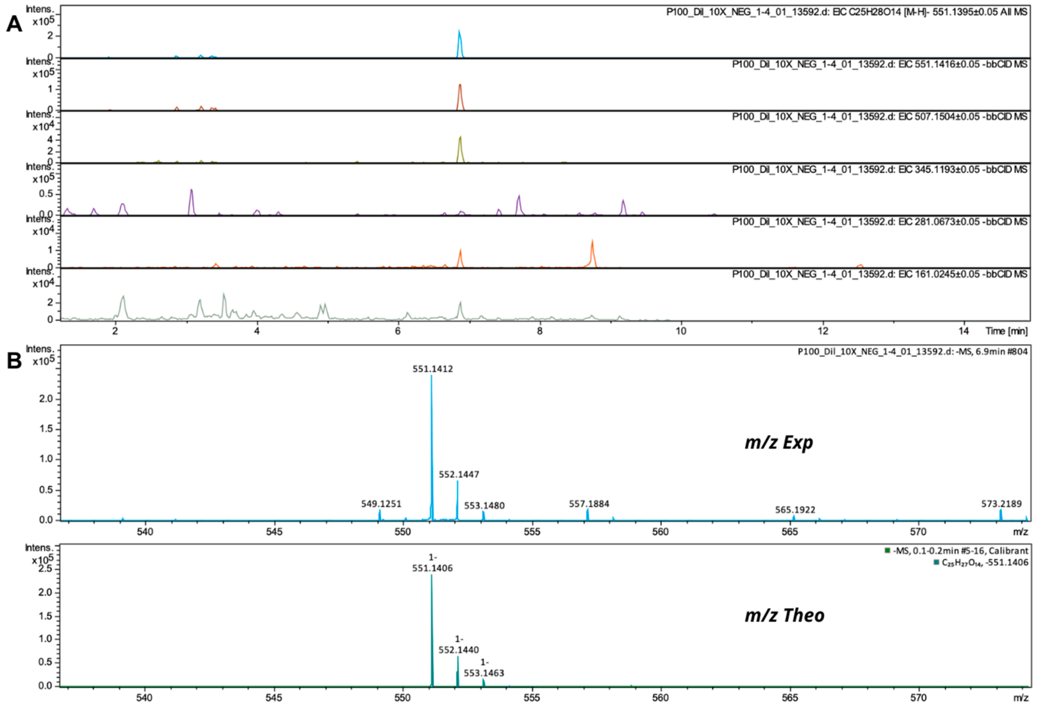

Figure S14. (A) MS/MS spectrum for caffeoyl-6'-secologanoside and its fragment ions; (B) Theoretical and experimental isotopic profile.

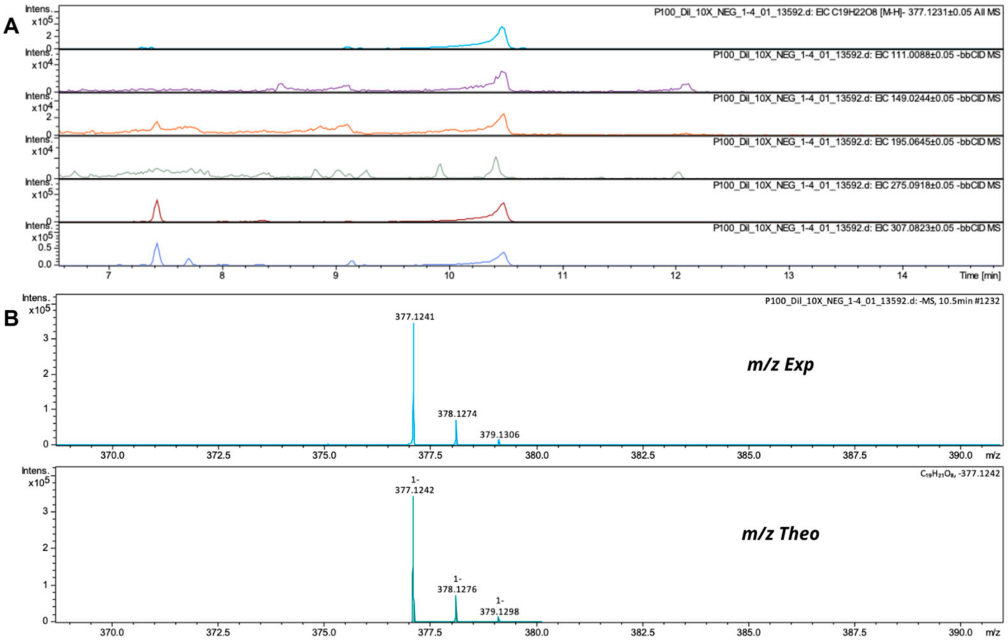

Figure S15. (A) MS/MS spectrum for oleuropein aglycone and its fragment ions; (B) Theoretical and experimental isotopic profile.

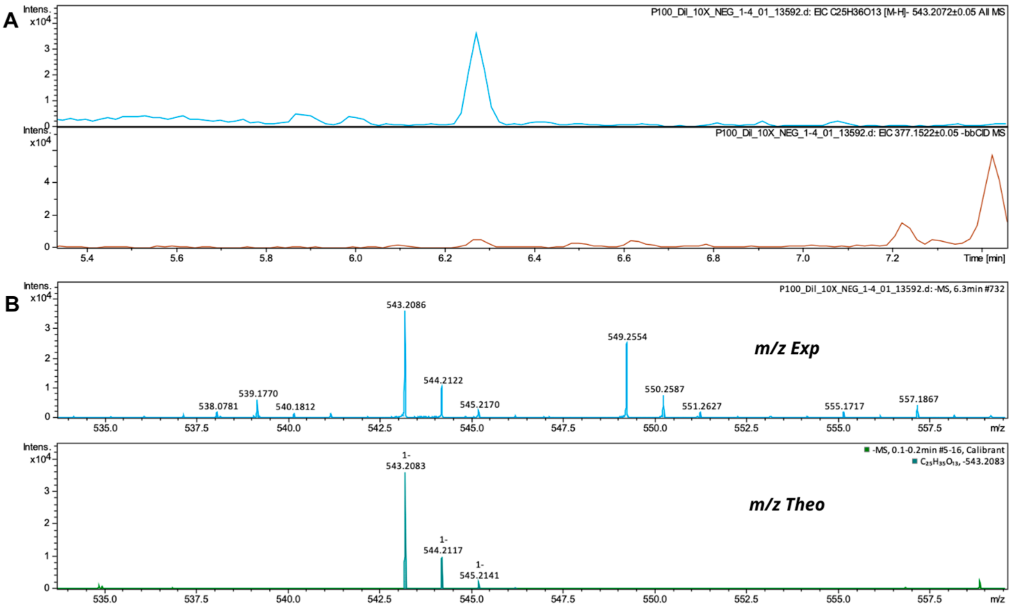

Figure S16. (A) MS/MS spectrum for dihydro-oleuropein and its fragment ions; (B) Theoretical and experimental isotopic profile.

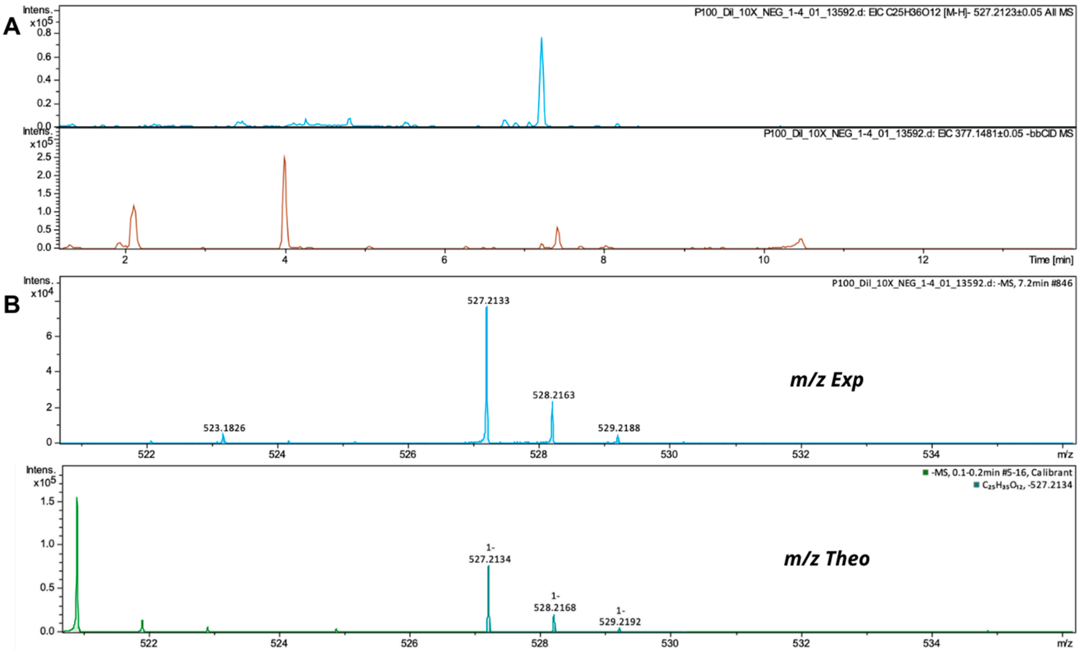

Figure S17. (A) MS/MS spectrum for oleuropein derivative and its fragment ions; (B) Theoretical and experimental isotopic profile.

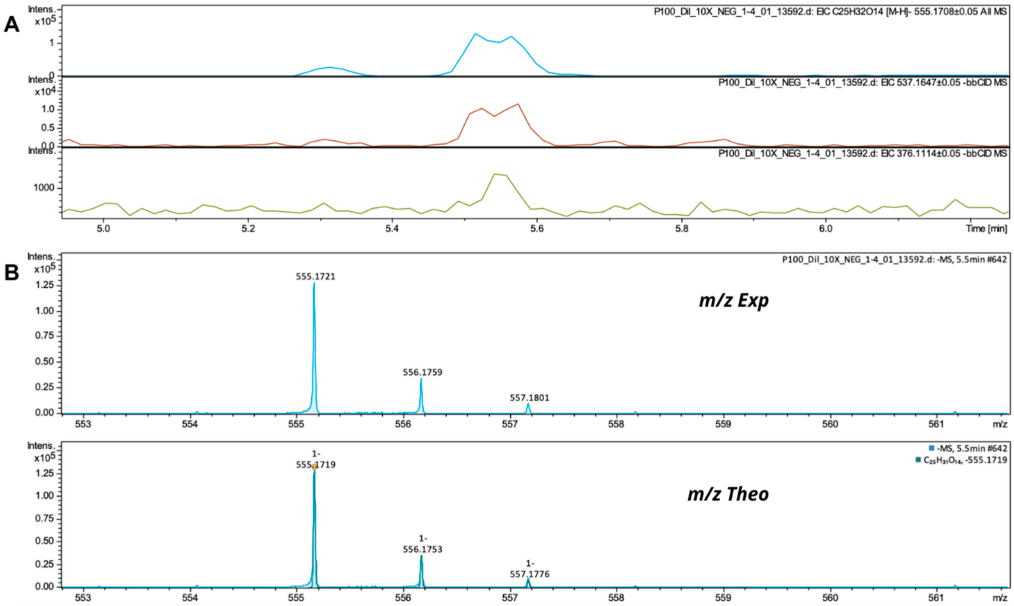

Figure S18. (A) MS/MS spectrum for 10-hidroxy-oleuropein and its fragment ions; (B) Theoretical and experimental isotopic profile.

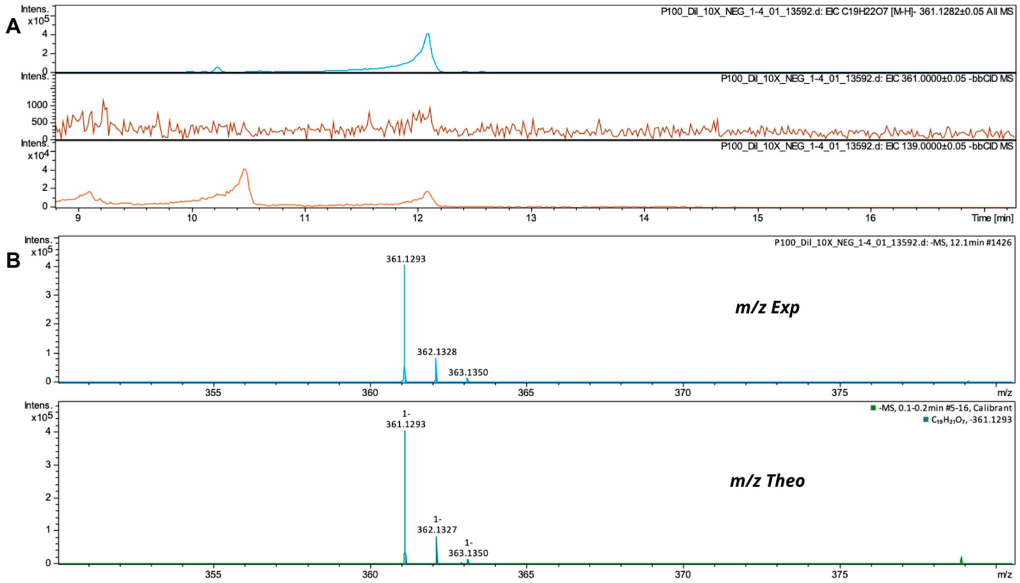

Figure S19. (A) MS/MS spectrum for p-HPEA-EA and its fragment ions; (B) Theoretical and experimental isotopic profile.

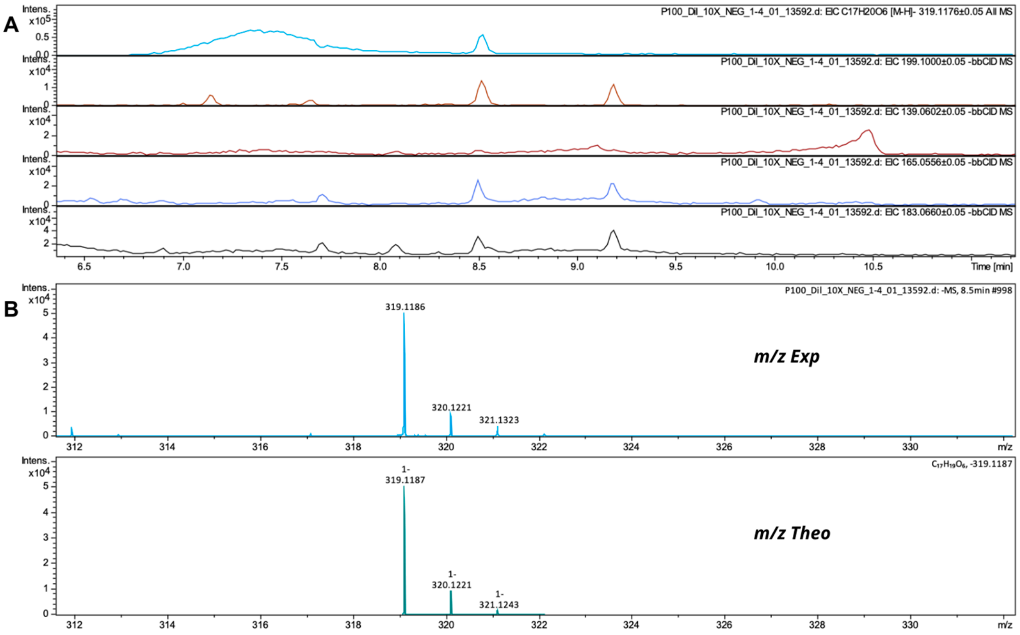

Figure S20. (A) MS/MS spectrum for oleacein and its fragment ions; (B) Theoretical and experimental isotopic profile.

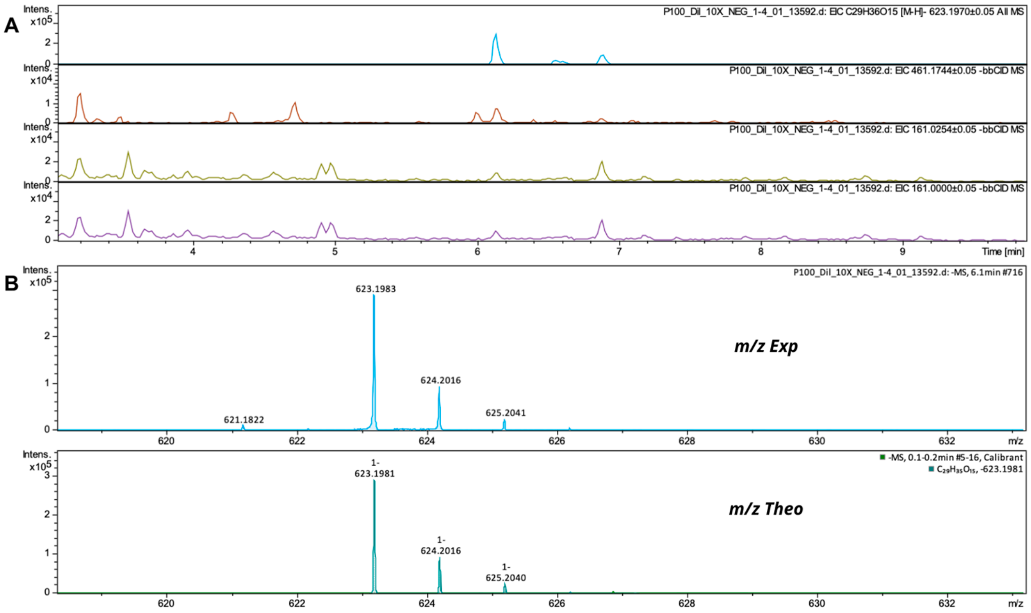

Figure S21. (A) MS/MS spectrum for verbascoside and its fragment ions; (B) Theoretical and experimental isotopic profile.

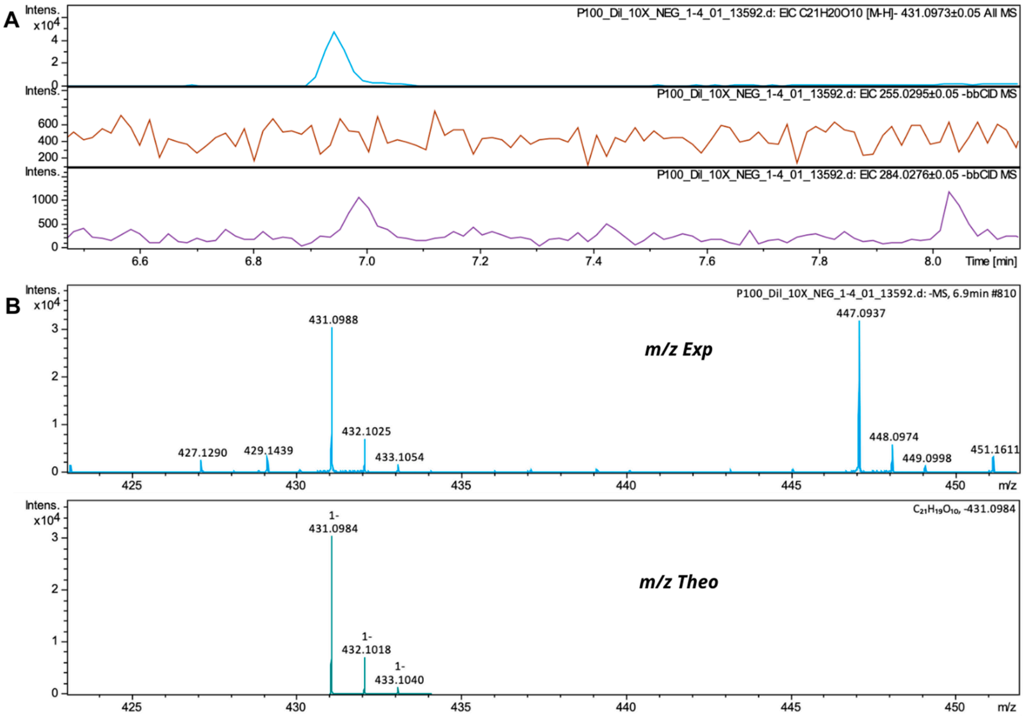

Figure S22. (A) MS/MS spectrum for luteolin and its fragment ions; (B) Theoretical and experimental isotopic profile.

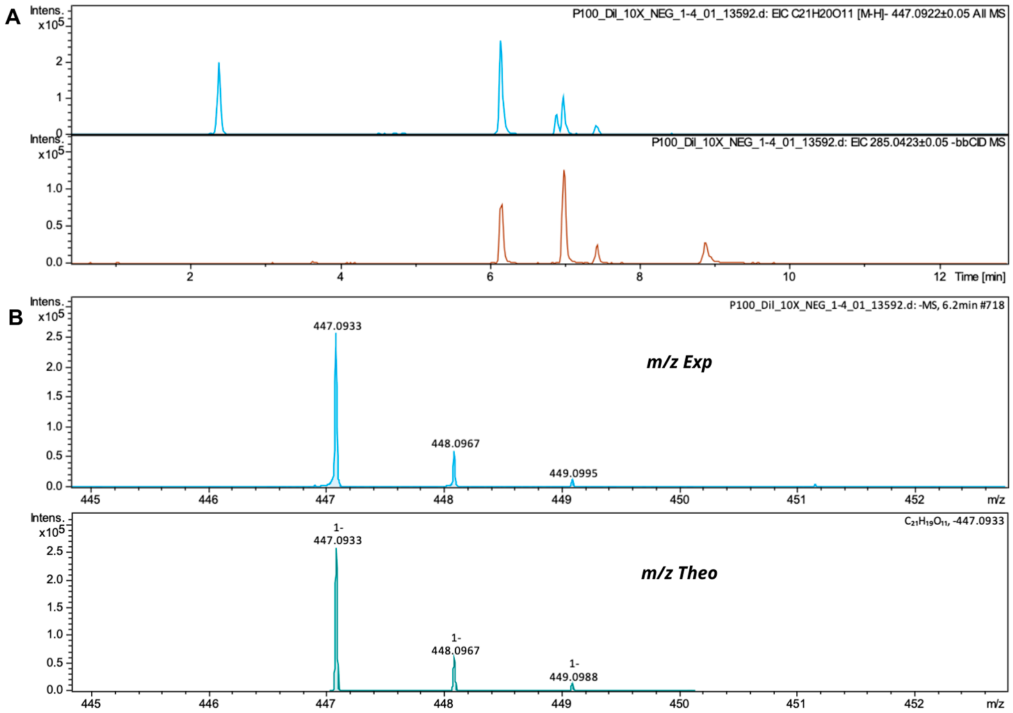

Figure S23. (A) MS/MS spectrum for luteolin glucoside and its fragment ions; (B) Theoretical and experimental isotopic profile.

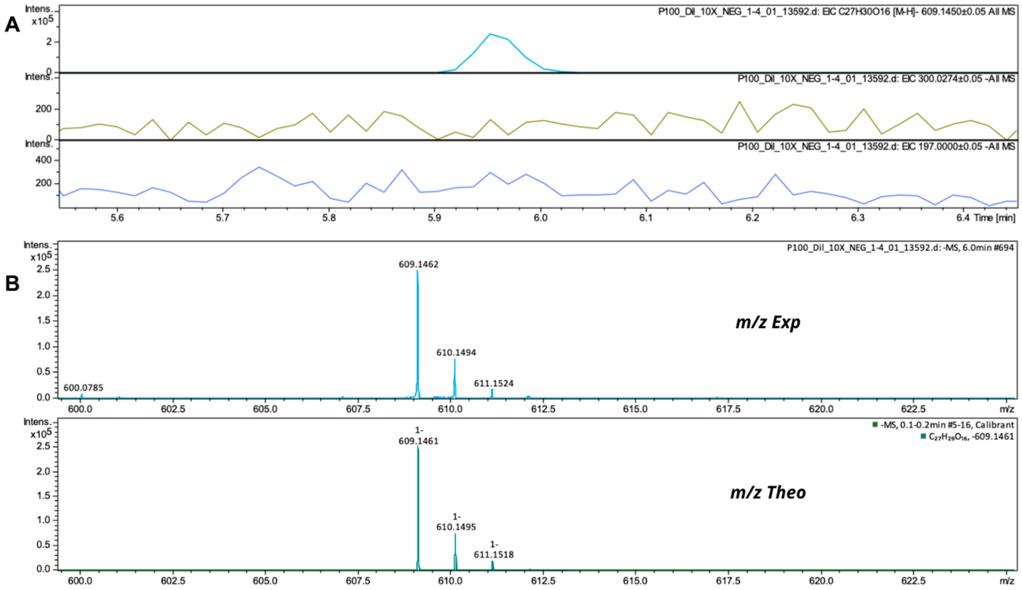

Figure S24. (A) MS/MS spectrum for rutin and its fragment ions; (B) Theoretical and experimental isotopic profile.

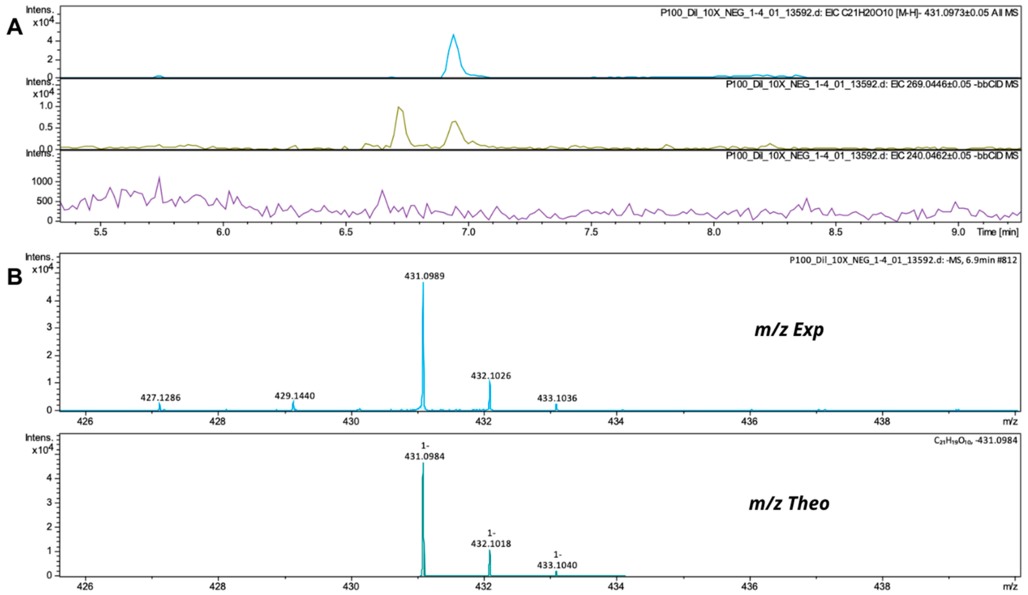

Figure S25. (A) MS/MS spectrum for apigenin glucoside and its fragment ions; (B) Theoretical and experimental isotopic profile.

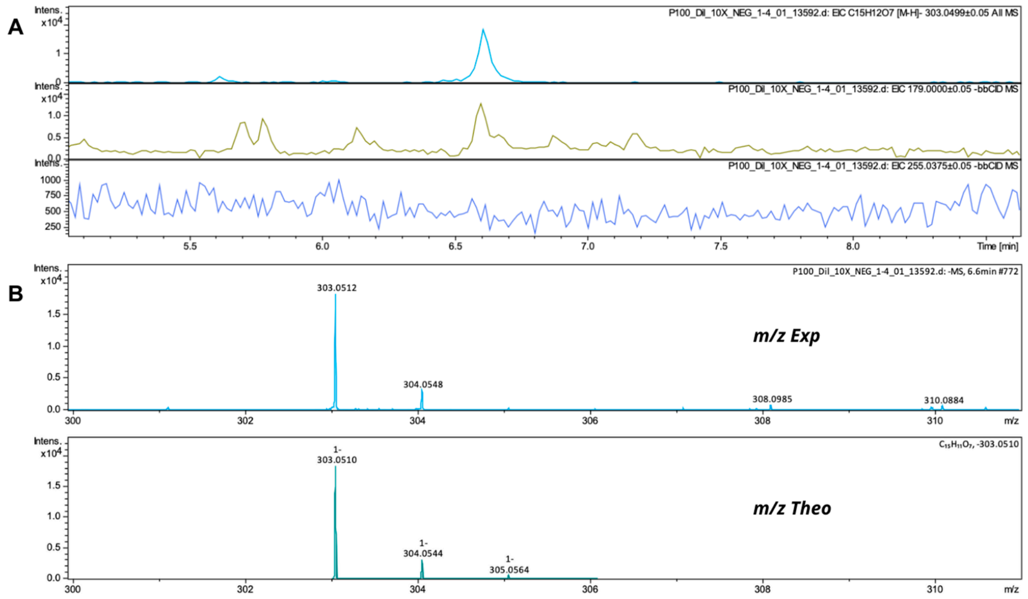

Figure S26. (A) MS/MS spectrum for taxifolin and its fragment ions; (B) Theoretical and experimental isotopic profile.

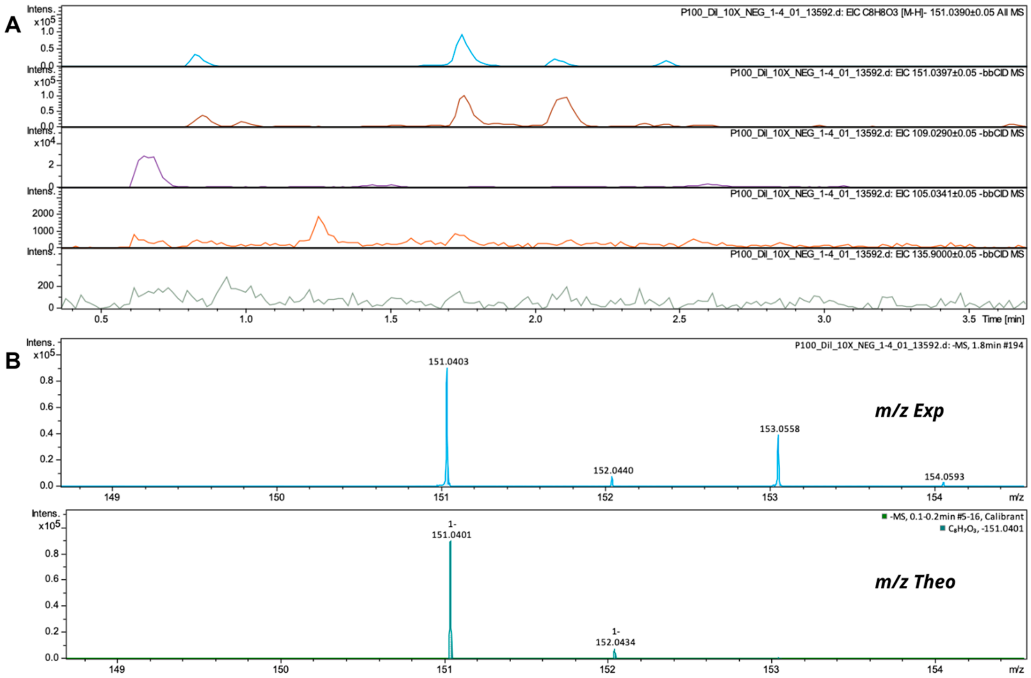

Figure S27. (A) MS/MS spectrum for vanillin and its fragment ions; (B) Theoretical and experimental isotopic profile.

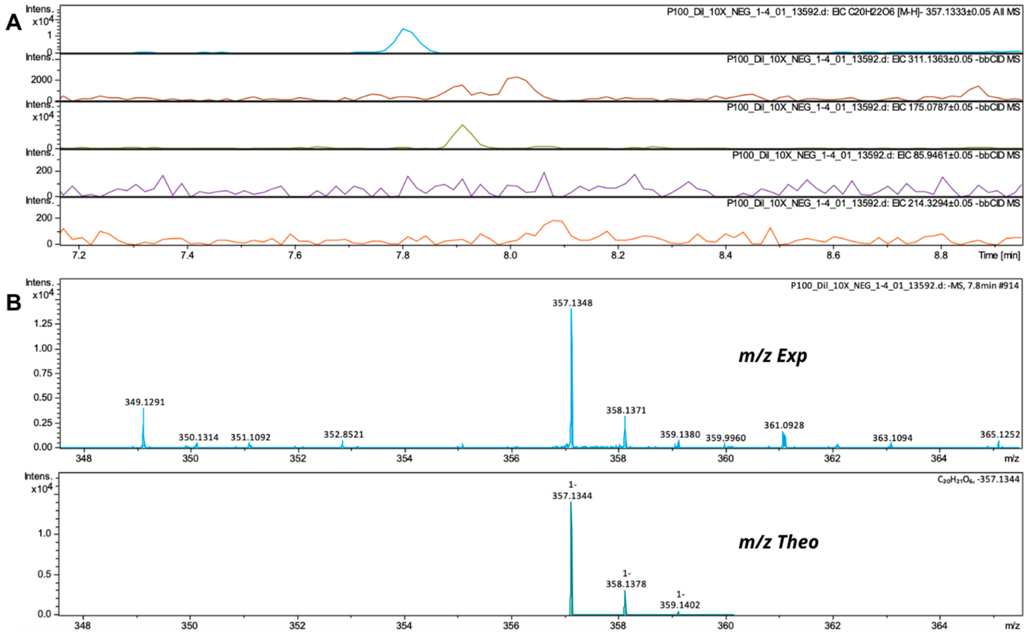

Figure S28. (A) MS/MS spectrum for pinoresinol and its fragment ions; (B) Theoretical and experimental isotopic profile.

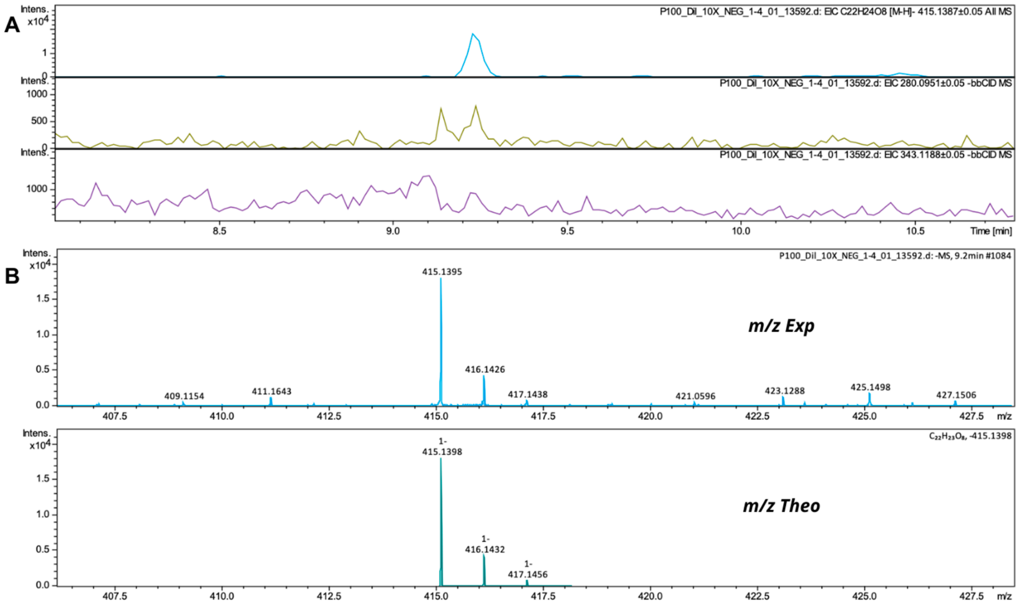

Figure S29. (A) MS/MS spectrum for acetoxypinoresinol and its fragment ions; (B) Theoretical and experimental isotopic profile.

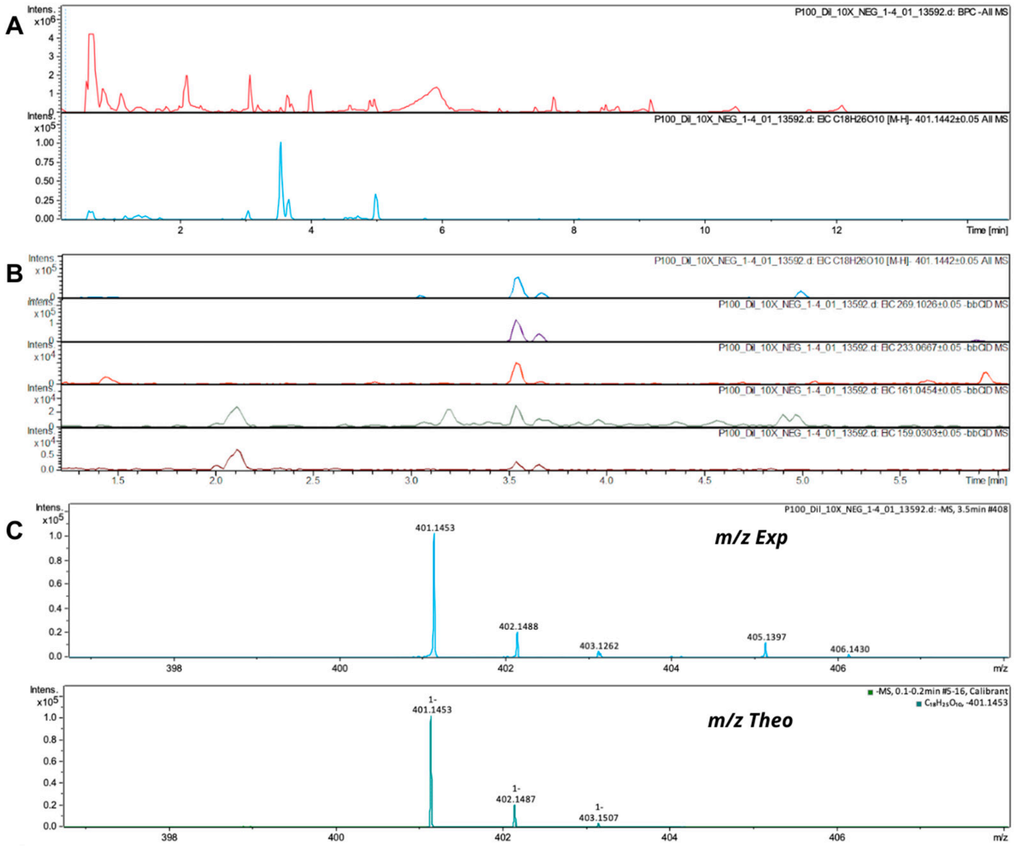

Figure S30. (A) MS/MS spectrum for benzyl primaveroside and its fragment ions; (B) Theoretical and experimental isotopic profile.
